# Defect Engineering Strategy for Superior Integration of Metal–Organic Framework and Halide Perovskite as a Fluorescence Sensing Material

**DOI:** 10.1021/acsami.4c00770

**Published:** 2024-04-23

**Authors:** Zhun-Xian Lai, Andi Magattang Gafur Muchlis, Ramadhass Keerthika Devi, Chen-Lung Chiang, Yi-Ting Syu, Yi-Ting Tsai, Cuo-Chi Lee, Chun Che Lin

**Affiliations:** † Institute of Organic and Polymeric Materials, National Taipei University of Technology, Taipei 106334, Taiwan; ‡ Research and Development Center for Smart Textile Technology, National Taipei University of Technology, Taipei 106334, Taiwan; § Department of Agricultural Science and Technology, Ministry of Agriculture, Taipei 100, Taiwan; ∥ Department of Biomedical Science, Chang Gung University, Taoyuan City 33302, Taiwan

**Keywords:** defect engineering, metal−organic framework, zeolitic imidazolate framework 90, halide perovskite, CH_3_NH_3_PbBr_3_ quantum dots, fluorescence sensing

## Abstract

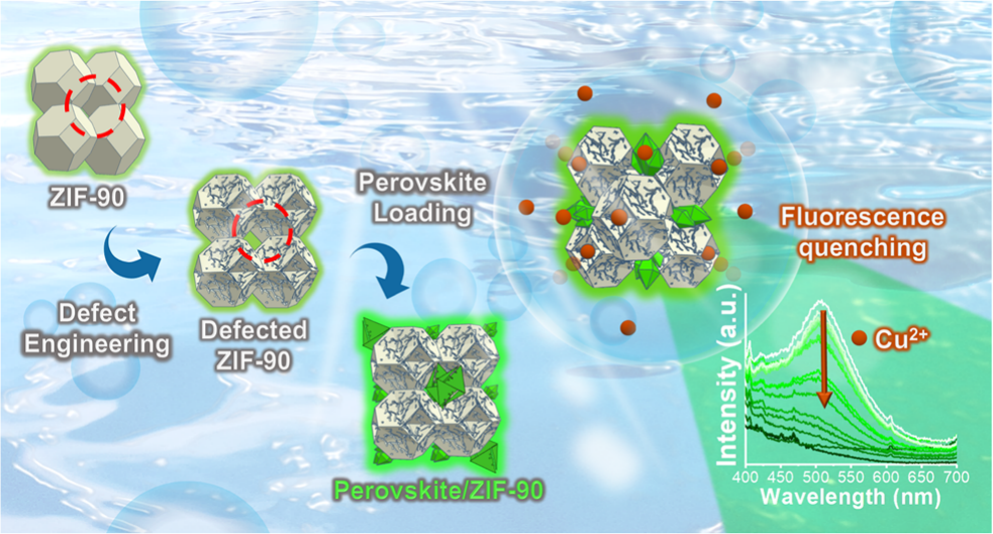

Combining halide perovskite quantum dots (QDs) and metal–organic frameworks (MOFs) material is challenging when the QDs’ size is larger than the MOFs’ nanopores. Here, we adopted a simple defect engineering approach to increase the size of zeolitic imidazolate framework 90 (ZIF-90)’s pores size to better load CH_3_NH_3_PbBr_3_ perovskite QDs. This defect structure effect can be easily achieved by adjusting the metal-to-ligand ratio throughout the ZIF-90 synthesis process. The QDs are then grown in the defective structure, resulting in a hybrid ZIF-90-perovskite (ZP) composite. The QDs in ZP composites occupied the gap of 10–18 nm defective ZIF-90 crystal and interestingly isolated the QDs with high stability in aqueous solution. We also investigated the relationship between defect engineering and fluorescence sensing, finding that the aqueous Cu^2+^ ion concentration was directly correlated to defective ZIF-90 and ZP composites. We also found that the role of the O–Cu coordination bonds and CH_3_NHCu^+^ species formation in the materials when they reacted with Cu^2+^ was responsible for this relationship. Finally, this strategy was successful in developing Cu^2+^ ion fluorescence sensing in water with better selectivity and sensitivity.

## Introduction

Copper (Cu^2+^) ions are commonly found in water. However, excessive amounts of Cu^2+^ ions in domestic water may cause human kidney and liver diseases. Thus, determining the presence of Cu^2+^ ions in domestic water is essential.^1−4^ Fluorescence sensing is widely used to detect specific metal ions, such as Cu^2+^ ions, because of its simplicity, convenience, and easy sample preparation.^5^ Some shortcomings of applying fluorescence sensing to detect trace metals, such as Cu^2+^, in water include an inadequate detection limit and a low selectivity toward Cu^2+^. Therefore, a more efficient, selective, and sensitive detection method for Cu^2+^ ions should be developed.

Metal–organic frameworks (MOFs) have attracted increasing research attention, and they have been used for various purposes, including gas sensing,^6,7^ molecular sieving,^8^ catalysis,^9^ electrical devices,^10^ and fluorescence sensing.^11−13^ An MOF is a three-dimensional structure that can be synthesized by using different metal ions and ligand combinations. MOFs are widely used as porous materials that have a large specific surface area and a fixed and controllable pore size and can easily be modified by altering the functional groups of their ligands. Zeolitic imidazolate framework 90 (ZIF-90) has been used for sensing because it possesses an ordered four- and six-membered ring pore structure, facilitates the coordination of metal ions with the aldehyde group of the ligand side chain, and exhibits fluorescent properties.^14−16^ The stability of the ZIF-90 structure by researchers has been utilized for supporting composites’ performance in some applications.^17,18^

The fluorescence sensing performance of MOFs such as ZIF-90 can be enhanced by combining them with superior photoluminescent materials, such as perovskites. Perovskites can have up to 90% photoluminescence quantum yield, a narrow fluorescent emission spectrum, and a freely adjustable visible light wavelength range. Thus, they have been used in photosensitive devices, quantum dot light-emitting diodes (QLEDs), and sensors.^19−27^ The perovskite structure is ABX_3_, which is composed of monovalent cations (A), divalent cations (B), and halide anions (X). Perovskite quantum dots (QDs) have a high degree of structural tolerance^19,20^ for not only all inorganic perovskites but also organometallic perovskites, such as CH_3_NH_3_PbBr_3_. CH_3_NH_3_PbBr_3_ contains the A-position cation CH_3_NH_3_^+^ that can provide crucial active sites for chemical reactions, especially when combined with MOFs and in sensing applications.^21,22^

Combining MOFs and perovskites could also become a solution for perovskites’ disadvantage regarding their instability toward water and moisture. Perovskite QDs typically survive for only 1 month at room temperature and are often applied in water. In other studies, the incorporation of perovskite into the interior of MOFs during the synthesis process limited the growth of perovskite, leading to the formation of MOFs/QDs composites that maintain the material’s fluorescence intensity for up to several months and maximize their applications.^28−34^

However, MOFs/QDs composite hybridization may still face difficulties, such as the size of the MOF pores used to load QDs being much smaller than that of the perovskite QDs. To overcome this problem, we demonstrated defect engineering toward the MOF structure; in this case, we used ZIF-90. Defected structures might give more space for more QDs’ loading. Several MOF structural defect types have been proposed, including ligand and metal ion defects^35^ and incomplete crystal stacking.^36^ A simple defect engineering can obtain all of these defect types during MOF synthesis by regulating their number of precursors (metals and ligands). Due to its simplicity, some physical and optical properties of crystal defects in MOFs have been extensively studied, and applications have been developed.^9,37,38^

Many studies in the field of defect engineering have proposed that increasing the defects of MOFs can improve the ion diffusion rate.^37^ However, using defect engineering to provide more perovskite growth sites in the MOFs can be an interesting novel approach to obtain better MOFs/QDs composite integration. Besides, no study in the field of fluorescence sensing has proposed the effect of changes in MOF crystal defects on the fluorescence quenching constant.

**Scheme 1:**
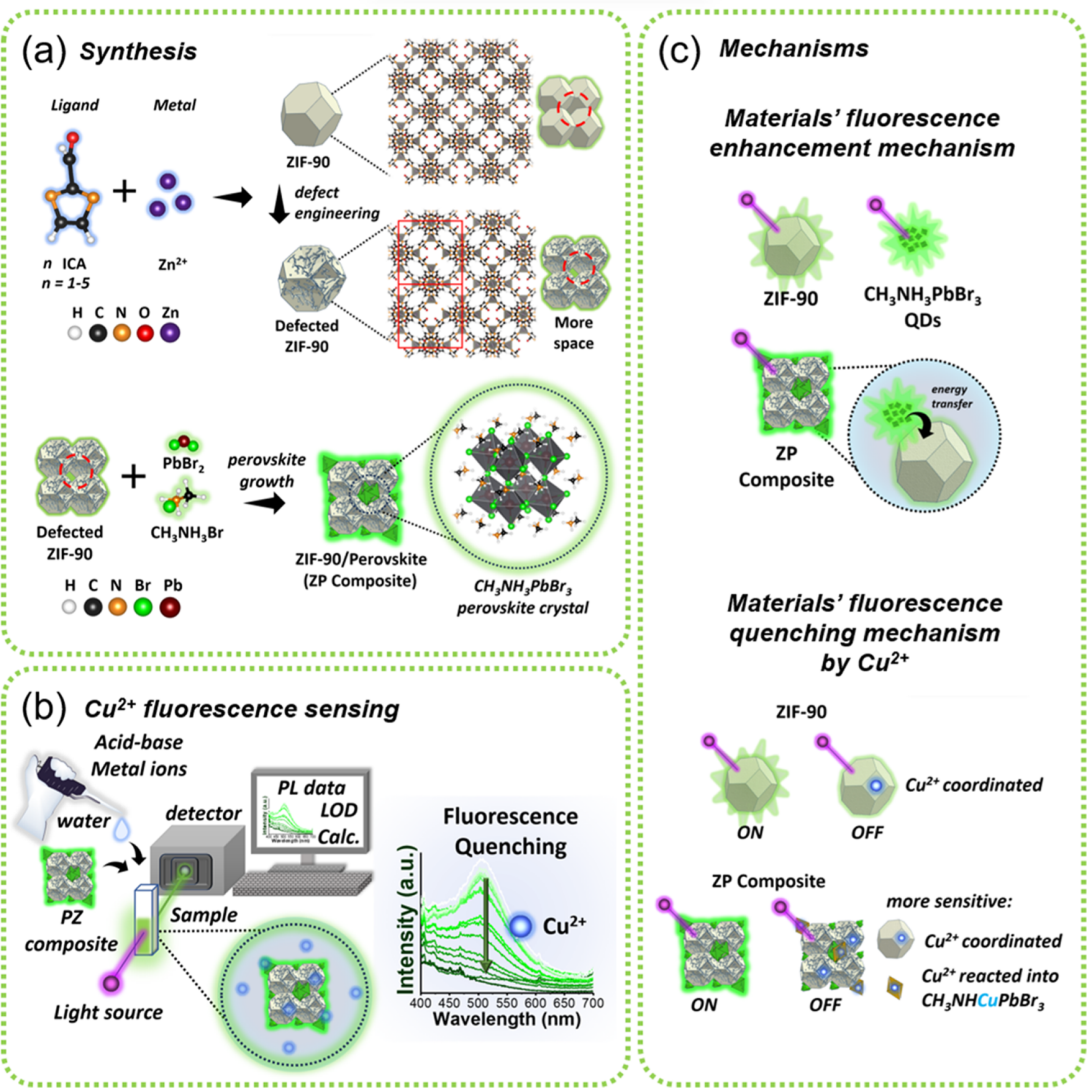
Schematic Illustration of the Research Concepts (a) Synthesis of ZIF-90 by using different ligand-to-Zn^2+^ mole ratios, followed by the preparation of ZP composites by adding CH_3_NH_3_Br and PbBr_2_; (b) Cu^2+^ fluorescence sensing in the water by prepared materials. (c) Schematic mechanism of materials fluorescence enhancement by CH_3_NH_3_PbBr_3_ as well as fluorescence quenching by Cu^2+^.

Therefore, in this study, we introduced defects in ZIF-90, an MOF with fluorescent properties, by varying the ligand-to-metal mole ratio (1–5:1) during the synthesis process. This defective engineering purpose is to provide more space to load more perovskite QDs. Subsequently, we combined those defected ZIF-90 with CH_3_NH_3_PbBr_3_ QDs by the addition of CH_3_NH_3_Br and PbBr_2_ to obtain improved luminescent materials (as described in Scheme 1a) for the highly selective sensing of Cu^2+^ ions in water via a fluorescence quenching mechanism once the material contacted with Cu^2+^ ions (Scheme 1b). As this material is applied in sensor application, acid–base and other metal ions assessment parameters are also taken to obtain a reliable result. We also provide a possible mechanism for material fluorescence enhancement and material fluorescence quenching by Cu^2+^ in Scheme 1c. Both ZIF-90 and CH_3_NH_3_PbBr_3_ QDs have luminescence properties in the green emission range. There are two possible mechanisms for the ZIF-90 fluorescence enhancement after combination with CH_3_NH_3_PbBr_3_ QDs. The first is because the green emission is mainly from the CH_3_NH_3_PbBr_3_ QDs. The second is energy transfer from CH_3_NH_3_PbBr_3_ QDs to ZIF-90. The designed ZIF-90/QDs composite material, named ZIF-90-perovskite (ZP) composite material in this paper, contained loaded perovskite QDs that were mainly located in the crystal stack of ZIF-90 and kept the QDs isolated from the aqueous solution but still allow Cu^2+^ ion transport through the ZIF-90 pore structures and chelated by ZIF-90 or/also bond with QDs forming oxidated products (CH_3_NHCuPbBr_3_). Both these mechanisms make the material lose its luminescence, thus causing fluorescence quenching. We examined the ion diffusion kinetics of the ZIF-90 pore size and the kinetic radius of Cu^2+^ ions. In addition, the effect of combining CH_3_NH_3_PbBr_3_ QDs on fluorescence sensing and the minimum sensing limits for different ligand-to-Zn^2+^ mole ratios were investigated. The results indicate that this method can effectively and sensitively sense Cu^2+^ ions in water.

## Experimental Section

### Reagents

All of the reagents were directly used without purification. We purchased 97% imidazole-2-carboxaldehyde (ICA), 98% Zn(NO_3_)_2_·6H_2_O, poly(vinylpyrrolidone) (PVP, molecular weight [*M*_W_] = 40,000), 99% dimethylformamide (DMF), 95% ethanol, ≥37% HCl, 99% NaOH, 99% NaCl, 99.5% KCl, 99% MgCl_2_, 97% CaCl_2_, >99% MnCl_2_, 99% NiCl_2_, 98% CuCl_2_, 97% ZnCl_2_, 98% HgCl_2_, and 99% PbCl_2_ from Alfa Aesar and 99% CH_3_NH_3_Br and 99.99% PbBr_2_ from Sigma-Aldrich.

### Synthesis of ZIF-90 by Using Different Ligand-to-Zn^2+^ Mole Ratios

ZIF-90 was synthesized as follows. First, two 50 mL vial bottles were labeled A and B, respectively. ICA (1.25, 2.50, 3.75, 5.00, or 6.25 mmol) and PVP (50 mg) were added to bottle A, followed by deionized water (25 mL). Then, the solution was heated to 70 °C and stirred at 300 rpm until precipitation of the solution was observed as the solution change became turbid with a yellowish brown color. Then, the solution was cooled to 40 °C and placed in a water bath for further use. Zn(NO_3_)_2_·6H_2_O (1.25 mol) and deionized water (3 mL) were added to bottle B. The solution was mixed evenly through stirring. The prepared solution B was then poured into solution A. The solution gradually became milky white, and the reaction time of the solution was maintained at 5 min. After 5 min, to prevent the continuous reaction of crystals and recrystallization, the reaction solution was centrifuged immediately to separate the precipitate. The precipitate was washed twice with deionized water (15 mL) and finally with ethanol (15 mL) once. Then, the precipitate was placed in a vacuum desiccator. After it dried, the precipitate was ground to powder and collected for use.^39^

### Synthesis of Micro-CH_3_NH_3_PbBr_3_

To a 50 mL vial were added CH_3_NH_3_Br (0.2 mmol) and PbBr_2_ (0.2 mmol), followed by DMF (5 mL). The formulated solution was placed in a shaker to ensure the complete dissolution of the precipitate before its removal. Then, toluene (20 mL) was added to the solution, and the supernatant in the reaction solution turned yellow. The supernatant was removed by centrifuging the solution at 7000 rpm for 3 min. The centrifuge tube was moved to a vacuum desiccator until the precipitate dried. Then, the dried precipitate was ground into powder, which was collected for further use.^19^

### Synthesis of ZP by Using Different Ligand-to-Zn^2+^ Mole Ratios

To a 20 mL vial was added the previously prepared ZIF-90 (80 mg), followed by CH_3_NH_3_Br (22.2 mg) and PbBr_2_ (73.4 mg). DMF (3 mL) and deionized water (2 mL) were added, and the solution was stirred for 1 h by using a magnetic stirrer at 300 rpm. The solution was filtered using a Büchner funnel to remove excess solution and precursors by washing with ethanol. The powder on the filter paper was collected with ethanol. Then, the solution was separated through centrifugation and placed in a vacuum desiccator until the precipitate dried. The dried precipitate was ground into powder, which was collected for further use.

### Fluorescence Sensing

The sample powder (80 mg) was added to deionized water (40 mL). The prepared solution was dispensed into different sample vials in 1 mL volumes, and the solution was shaken before each dispensing to ensure that the powder in the solution was evenly dispersed in the aqueous solution. The solution was moved to a quartz cuvette, and the standard solution was added. After 1 min of reaction, the photoluminescence fluorescence spectrum was determined, and the measurement slit was adjusted to 2 nm.^12^

### Material Characterization

X-ray diffraction (XRD) [PANalytical X’Pert3 Powder, Cu Kα source (1.541 Å), The Netherlands] was used to analyze the crystal structure. Scanning electron microscopy (SEM) [HITACHI S-4800, 15 kV, Japan] and high-resolution transmission electron microscopy (HRTEM) [JEOL JEM-2100F, 200 kV, Japan] were performed to analyze the crystal surface morphology. Inductively coupled plasma-optical emission spectroscopy (ICP-OES) [PerkinElmer Optima 8000] was used to analyze Zn and Pb percentages in the composites. The Brunauer–Emmett–Teller (BET) method was used to analyze nitrogen adsorption and pore size distribution using a specific surface area and pore size analyzer [Micromeritics TriStar II PLUS]. X-ray photoelectron spectroscopy (XPS) [VG Scientific ESCALAB 250, Twin anode X-ray gun, 15 kV, United Kingdom] and ultraviolet (UV) spectrometry [Jasco V-770, Japan] were performed to analyze the material bonding energy and material absorption wavelengths, respectively. Photoluminescence spectroscopy (PL) [HORIBA Jobin Yvon, Japan] was used to measure the fluorescence spectral range. Crystal models were drawn by using VESTA software.

## Results and Discussion

### Material Structure Analysis

**Figure 1:**
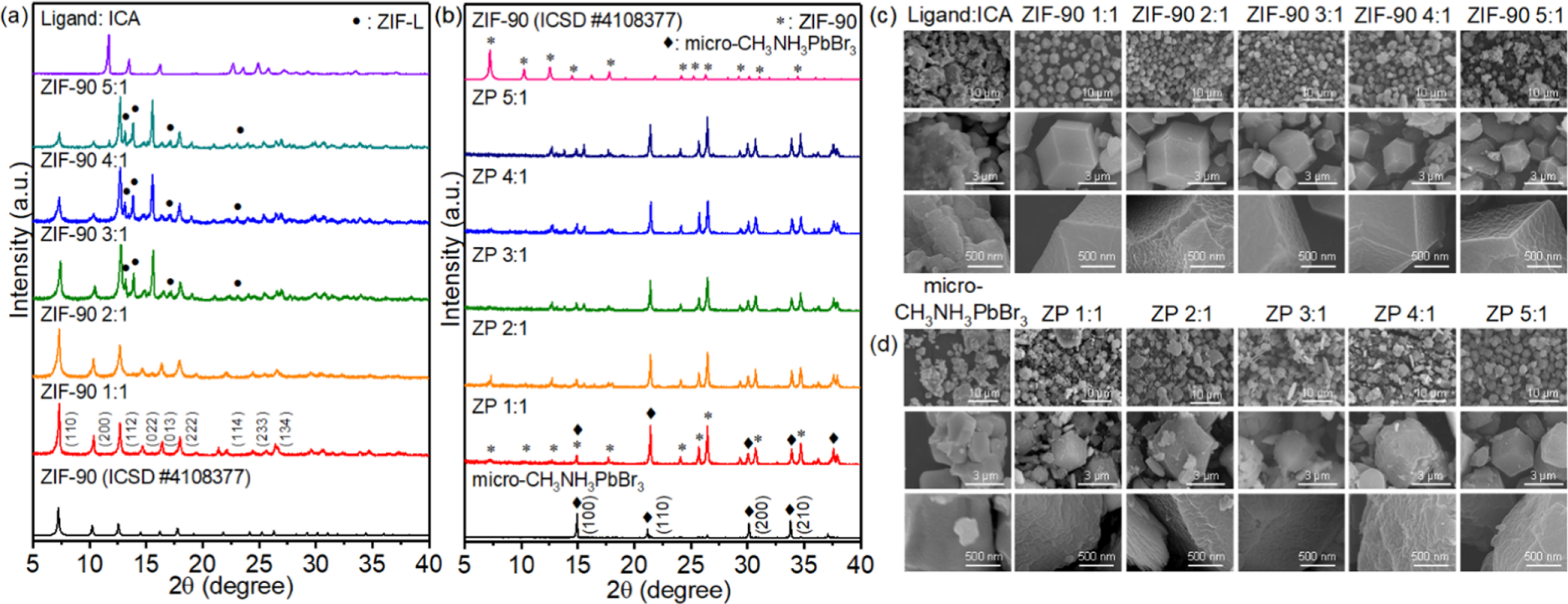
X-ray diffraction (XRD) analysis of (a) ZIF-90 and (b) ZP in different ligand-to-Zn^2+^ mole ratios. SEM images of (c) ZIF-90 and (d) ZP.

The addition of zinc ions to ICA led to the formation of the ZIF-90 structure, and the ZIF-90 structure prepared using different ligand-to-Zn^2+^ mole ratios was analyzed by using XRD (Figure 1a). Compared with the standard XRD pattern of ZIF-90, all ZIF-90 with different ligand-to-Zn^2+^ mole ratios well matched with main peaks from low-angle to high-angle crystal planes in the order of (011), (200), (112), (022), (013), (222), (114), (233), and (134).^13−16,39,40^ This finding indicates that the synthesis process was appropriate and that all of the resulting materials were successfully prepared. When the ratio exceeded 2:1, ICA in the solution could not completely dissociate the H^+^ ion, resulting in the linkage of the Zn^2+^ ion and ICA. A formaldehyde coordination reaction occurred, and hydrogen bonds promoted the molecular arrangement to form the ZIF-L structure (Figure S1).^41,42^ The structure of ZP composites prepared using different ligand-to-Zn^2+^ mole ratios was analyzed by using XRD (Figure 1b). The crystalline phase of CH_3_NH_3_PbBr_3_ existed in the composite material, and its crystalline peak revealed the order of (100), (110), (200), and (210). When the mole ratio was higher, the crystallinity of perovskites was lower, indicating that the number of perovskites decreased gradually.^28−30,33,34,43,44^

SEM images were used to analyze the morphology of these prepared ZIF-90 materials (Figure 1c). The surface morphology of ZIF-90 indicated that the crystal is a dodecahedron. Its size gradually decreased with an increase in the mole ratio, mainly because of the effect of secondary nucleation,^45^ resulting in a smaller-sized crystal distribution. At a mole ratio of >2:1, irregular and aggregated ZIF-L crystals were observed. On the other hand, ZP composite morphology was also subsequently analyzed (Figure 1d). We noted excess micro-CH_3_NH_3_PbBr_3_ crystals around ZIF-L crystals; this finding was attributed to crystal aggregation, leading to the precursor remaining around ZIF-L.

### Perovskite Growth Sites

To determine whether the position occupied by perovskite QDs affects the diffusion of Cu^2+^ ions in water, the perovskite should occupy the crystal position of ZIF-90. First, the structures of ZIF-90 and ZIF-L are three- and two-dimensional, respectively. The ZIF-90 structure can be divided into a cavity of 11 Å, a six-membered ring of 3.5 Å, and a four-membered ring of 2.2 Å (Figure S2).^14−16^ By contrast, ZIF-L is a nonporous structure.^41,42^ Increased ZIF-L phase reduced the ability of ZIF-90 to accommodate QDs.

**Figure 2:**
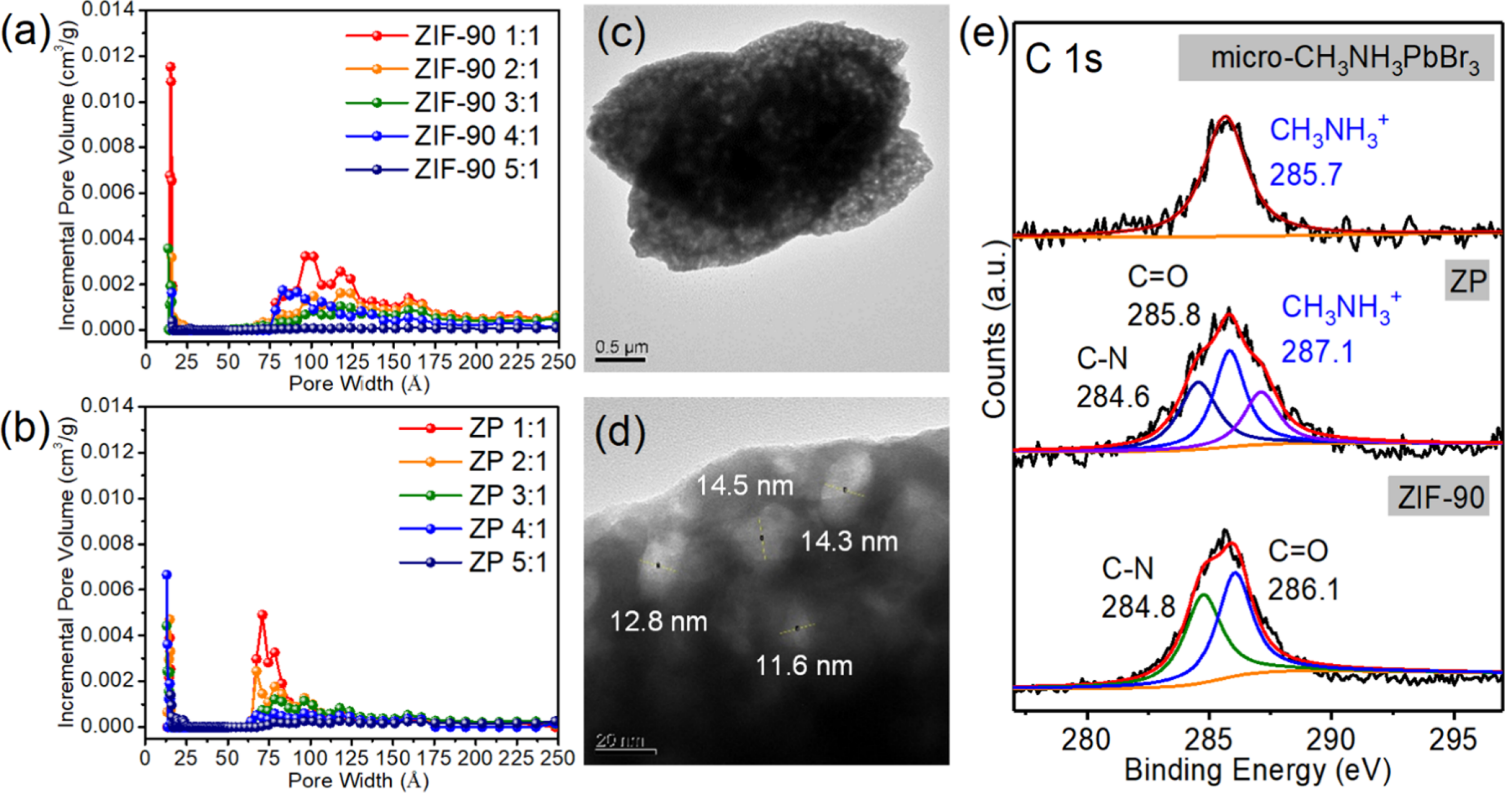
Brunauer–Emmett–Teller (BET) analysis of (a) ZIF-90 and (b) ZP. HRTEM images of ZP with typical (c) low magnification and (d) high magnification. (e) XPS spectra of the materials.

The pore size distribution of ZIF-90 prepared using different ligand-to-Zn^2+^ mole ratios was observed (Figure 2a). The 2 nm micropores referred to pores present in the ZIF-90 crystal structure, whereas 6 to 18 nm mesopores referred to pores caused by ligand linker vacancies and crystal growth defects.^35,36,38^ The pore volume gradually decreased with an increase in the ligand ratio. Figures S3 and S4 present the complete diagram of nitrogen adsorption–desorption for ZIF-90 and ZP composites, revealing the specific surface area of these materials. A higher ligand concentration reduced the number of vacant linkers of the ligand and promoted crystal growth, decreasing the number of the two types of defects.^8,9,12^

We also evaluated the pore size distribution of the ZP composites (Figure 2b). The distribution of mesoporous structures ranged from 6 to 18 nm, and the pore size distribution from 10 to 18 nm was not observed. This finding indicates that CH_3_NH_3_PbBr_3_ QDs occupied the MOF’s 10 to 18 nm pore structure. Energy-dispersive spectroscopy (EDS) analysis revealed that QDs successfully occupied ZIF-90 pores, indicating evenly distributed perovskite elements in the ZIF-90 structure (Figures S5–S8).

To confirm the occupied position of CH_3_NH_3_PbBr_3_ QDs, we performed HRTEM analysis. The HRTEM images of ZIF-90, ZP composites, and micro-CH_3_NH_3_PbBr_3_ as well as their elemental distribution are presented in Figures S9–S11 and Tables S1–S3, respectively. HRTEM indicated that the structure of micro-CH_3_NH_3_PbBr_3_ was cracked because of the high energy of the electron beam (Figure S10)^46^ and then decomposed into PbBr_2_. The HRTEM analysis indicated that the Pb-to-Br atomic percentage ratio in micro-CH_3_NH_3_PbBr_3_ was 1:2 (Table S3). We determined whether this ratio conformed to the pore size distribution by examining the remaining pore size.

Figure 2c,d presents the HRTEM images of the ZP composite (3:1) at different magnifications. At high magnification (Figure 2d), some vacant holes in the composite structure ranged from 11.6 to 14.5 nm, indicating that QDs grew around that size and then were decomposed by high-energy HRTEM. In other words, vacancies of crystal defects exhibited the perovskite growth site in these materials. Figures S12 and S13 show TEM images of ZIF-90 with different ratios and HRTEM of ZP composites, respectively. Figure S12 has a pattern similar to that of HR-SEM data. However, the porous surface for each sample of ZIF-90 cannot be clearly observed. On the other hand, in Figure S13, we can observe that, by increasing the ligand–metal ratio, the vacant holes (caused by QDs growth) also increased in amount and size until the ratio of 4:1. Due to the formation of ZIF-L in the sample 5:1, the formation of micro-CH_3_NH_3_PbBr_3_ outside of the ZIF-90 structure; thus, the vacant holes cannot be observed significantly.

To support our assumption that increasing the ZIF-90 ligand–metal ratio could load more perovskite QDs, we took ICP-OES to evaluate Pb (representing CH_3_NH_3_PbBr_3_ perovskite QDs). The data are delivered in Figure S14. These data confirm the concentration of Pb is relatively increasing in line with the higher ligand-to-Zn^2+^ ratio from 1:1 to 4:1 (reach 51.44 wt %). As we mentioned, for 5:1, it became slightly reduced as the formation of ZIF-L, thus reducing the capability to load more perovskite QDs.

XPS analysis revealed that the binding energy peaks of the ZP composite fit with those of ZIF-90 and micro-CH_3_NH_3_PbBr_3_, indicating successful preparation of the ZP composite. The complete XPS spectra of these materials and their detailed signals, such as C 1s, N 1s, O 1s, Zn 2p, Pb 4f, and Br 3d, are presented in Figures S15–S18. When the ZP composite formed, ZIF-90 interacted with CH_3_NH_3_PbBr_3_ QDs because of the interaction of the A-site cation CH_3_NH_3_^+^ with the imidazolyl bond, resulting in changes in these binding energy signals (Figure 2e).^43,44^

### Materials Optical Properties

The observed UV–visible (UV–vis) absorption spectrum of ZIF-90 was divided into two absorption sites (Figure S19): the 369 nm formaldehyde group and the 449 nm imidazolyl group.^13^ The lowest excited state of the highest occupied molecular orbital (HOMO) to the lowest unoccupied molecular orbital (LUMO) was determined by calculating the lowest excited state of the imidazolyl group (Figure S20).^47^ The formaldehyde group was almost unchanged, mainly because of the ligand defect generated by the vacancy link, wherein the positive charge of the Zn^2+^ ion interacted with the electron, causing a decrease in the lowest excited state of the defective crystal.^9^ In other words, when the ligand-to-Zn^2+^ ratio increased, ligand defects gradually decreased and the bandgap gradually increased.

Analysis of the ZP composite UV–vis absorption spectrum (Figure S21) indicated the presence of the absorption band of CH_3_NH_3_PbBr_3_ QDs at 369 nm. The ZP composite and ZIF-90 imidazolyl group exhibited the same change in energy (Figure S22).^29,43,48^ When irradiated with an excitation wavelength of 369 nm, the maximum emission wavelength of ZIF-90 is 490 nm and the maximum emission wavelength of ZP is 515 nm. To study the effect of QDs addition to ZIF-90, we observed ZIF-90 excitation wavelengths at an emission of 515 nm too.

**Figure 3:**
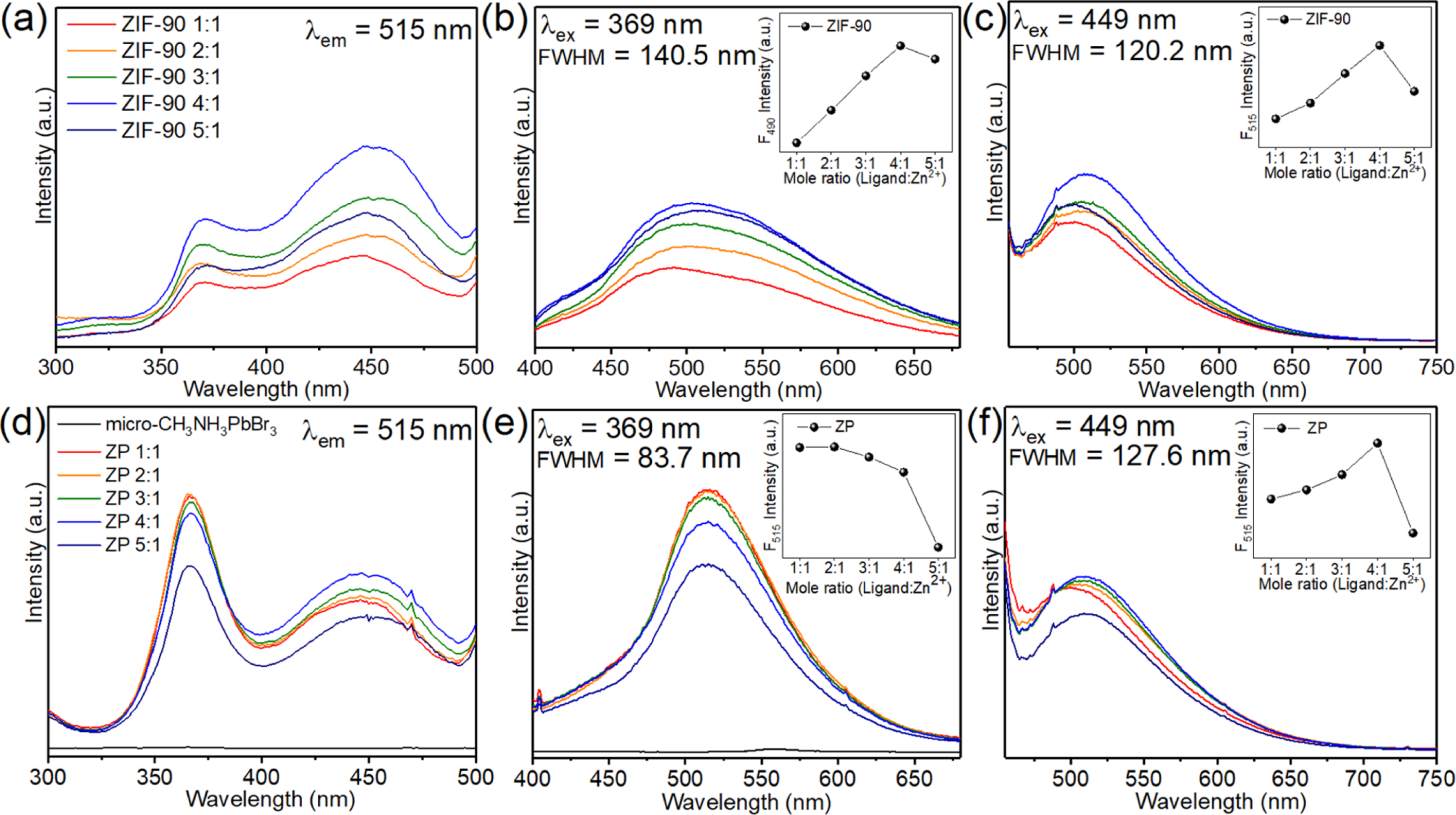
PL analysis of ZIF-90 and ZP materials. (a) Excitation spectra of ZIF-90; (b) 369 nm of emission spectra and (c) 449 nm of emission spectra. (d) Excitation spectra of ZP; (e) 369 nm of emission spectra and (f) 449 nm of emission spectra.

The ZIF-90 photoluminescence (PL) spectrum exhibited two excitation wavelengths (Figure 3a), corresponding to the 369 and 449 nm absorption sites, respectively. The ligand-to-Zn^2+^ mole ratio of 4:1 resulted in the highest fluorescence intensity. When ligand defects gradually decreased, the fluorescence intensity gradually increased. When the number of ligand defects decreased, the fluorescence intensity decreased because of ZIF-L formation. The full width at half-maximum (fwhm) of the 369 and 449 nm emission spectra was 140.5 (Figure 3b) and 120.2 nm (Figure 3c), respectively. A higher excitation HOMO resulted in a wider half-width of ZIF-90 under a 369 nm excitation light source.

On the other hand, we also measured the excitation spectra of ZP materials (Figure 3d). The ratio of the 369/449 nm peak relative intensity in ZP composites increased to 1.33 compared to ZIF-90, which was only 0.63, indicating that CH_3_NH_3_PbBr_3_ QDs addition affected the excitation spectra and further enhanced emission intensity. The experimental design in this study is to use the crystal of ZIF-90 as the growth template of the CH_3_NH_3_PbBr_3_ QDs. So, the ZP composite material and micro-CH_3_NH_3_PbBr_3_ synthetic method should be similar. By this method, we can only produce micro-CH_3_NH_3_PbBr_3_, and we used it as a comparison material because of its structure being identical to its QDs in the ZP composite.

However, because the crystal size of micro-CH_3_NH_3_PbBr_3_ is larger than 100 nm, it will not emit light.^20^ The fwhm of the 369 nm emission spectrum for ZP was 83.7 nm (Figure 3e). When the mole ratio of the ligand was higher, the fluorescence intensity gradually decreased, mainly because the number of CH_3_NH_3_PbBr_3_ QDs gradually decreased and the number of micro-CH_3_NH_3_PbBr_3_ gradually increased. The fwhm of the 449 nm emission spectra was 127.6 nm (Figure 3f), which corresponded to the fluorescence intensity of ZIF-90, indicating that CH_3_NH_3_PbBr_3_ QDs did not emit fluorescence at 449 nm.

When CH_3_NH_3_PbBr_3_ grew in the ZIF-90 crystal and produced ZP, the size of the CH_3_NH_3_PbBr_3_ crystal was confirmed by BET and TEM as 10 to 18 nm. In that small size, electrons would be confined when electrons were excited by the energy because the surface energy was too high, resulting in an enhanced quantum confinement effect and further enhanced luminous effect with narrower fwhm. The fluorescence emission spectrum shows that the fwhm at 369 nm was reduced to 83.7 nm, but the fwhm at 449 nm was 127.6 nm, which hardly changed. These results indicated that CH_3_NH_3_PbBr_3_ in ZP was nanosized and might use 369 nm excitation light to emit high fluorescence intensity. Moreover, according to the literature, CH_3_NH_3_PbBr_3_ will gradually approach 530 nm when the size is reduced, and CH_3_NH_3_PbBr_3_ has been confirmed to have about 90% external quantum efficiency.^28−30,33,34,44^

So far, our investigation of PL enhancements in the excitation of 369 nm might be caused by two possible mechanisms. The first one is that adding QDs enhances the better luminous effect by decreasing QDs nanosize (10–18 nm). The second involves energy transfer to enhance the luminous ability of ZIF-90. By QDs emission and ZIF-90 absorption spectral comparison, we can estimate the possible mechanism of PL enhancements as shown in Figure S23. The emission spectrum of QDs is a little bit overlapped with the edge of absorption spectra of ZIF-90. Energies might transfer from QDs to ZIF-90, resulting in ZIF-90 exhibiting higher PL intensity by QDs support.^49,50^ However, based on the emission spectra at excitation of 449 nm with no significant change in fwhm (see Figure 3c,f), the energy transfer mechanism in ZP might have a smaller role. In other words, QD emissions in ZP are more dominant than ZIF-90 emissions.

### Fluorescence Sensing Experiment

To investigate the effect of adding CH_3_NH_3_PbBr_3_ QDs, 369 nm was selected as the excitation wavelength of fluorescence sensing and spectral changes between ZIF-90 and ZP composites were compared. The fluorescent stability of material for fluorescence sensors is essential. Therefore, we investigated ZIF-90 and ZP composite fluorescence intensity in water by time, and we found that both were stable in the water after 10 min (Figures S24 and S25). Because each PL analysis sample in this work reaction procedure is not more than 1 min, with this stability, these materials are applicable for sensing in water.

**Figure 4:**
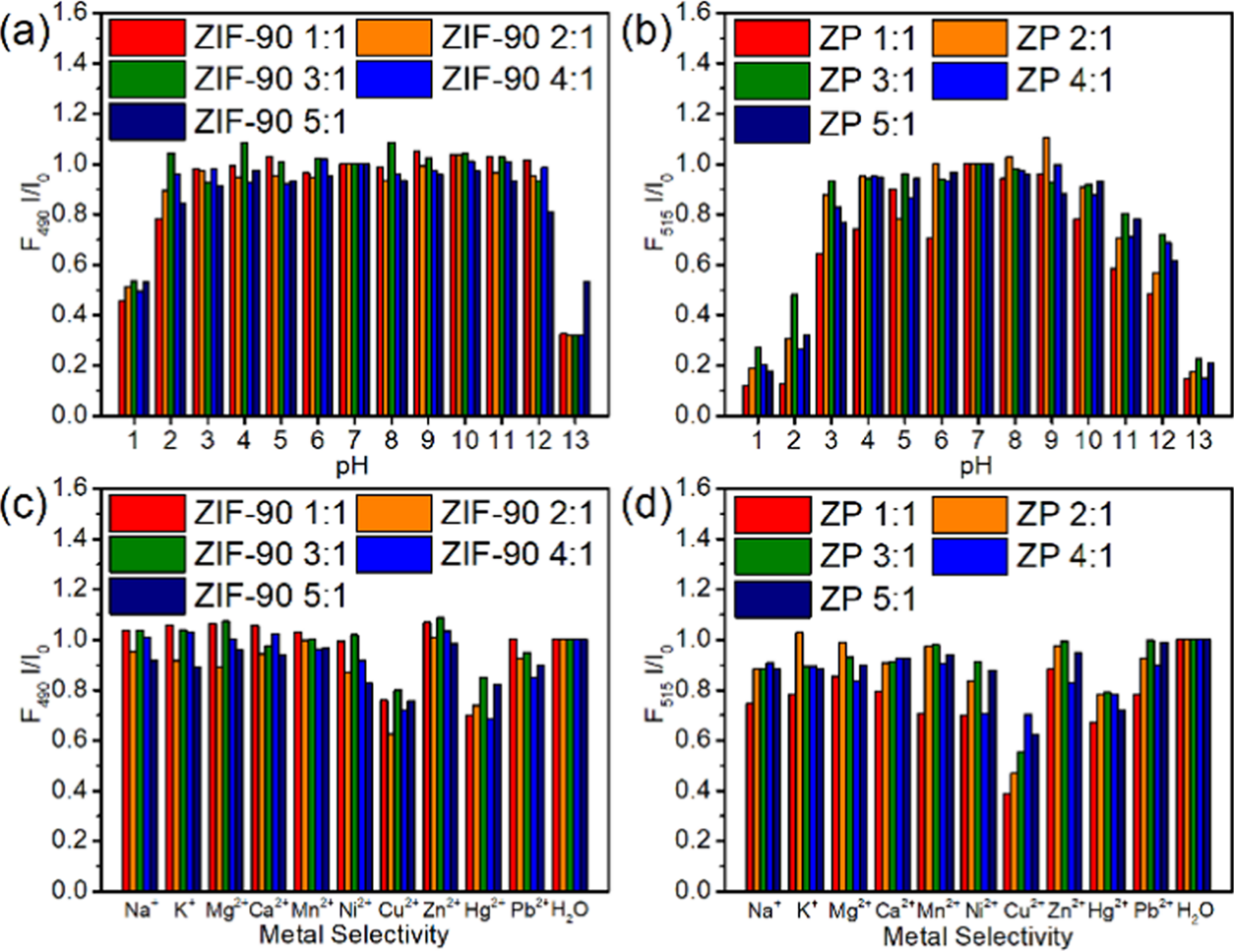
Acid–base tolerance test of (a) ZIF-90 and (b) ZP composites in Cu^2+^ sensing. Metal ion selectivity test of the (c) ZIF-90 and (d) ZP composites.

Moreover, because the pH of the salt solution in water would differ, the two materials with different ligand-to-Zn^2+^ mole ratios were added to the acid–base standard solution to measure the fluorescence emission spectrum (Figures S26 and S27). Although the spectra of the two materials did not shift, we noted a change in the intensity because of the decomposed composite structure. We calculated the average relative fluorescence intensity (Tables S4 and S5). The average fluorescence relative intensity of ZIF-90 prepared using different ligand-to-Zn^2+^ mole ratios maintained more than 80% of the fluorescence effect at pH 2–12 (Figure 4a). The average fluorescence relative intensity of ZP composites prepared using different ligand-to-Zn^2+^ mole ratios maintained more than 80% of the fluorescence effect at pH 3–10 (Figure 4b). In addition, the decline in stability under the acid–base condition may be due to the decrease in bonding energy caused by the interaction between ZIF-90 and CH_3_NH_3_PbBr_3_ QDs.^51,52^

Considering that the pH values of salt aqueous solutions with the same molar concentration differ, we measured the pH value of the salt by using a pH meter (Table S6). All solutions were within the pH range of ZIF-90 and ZP composite materials. Other anions and materials were avoided to ensure that other reactions would not occur. Chloride salts were selected in the metal selectivity test, and the fluorescence emission spectra of the metal ion solutions were analyzed (Figures S28 and S29). No shift was observed in the spectra of the two materials. Analysis of the relative intensity of the average fluorescence revealed that ZIF-90 and ZP composites exerted a stronger fluorescence quenching effect on the Cu^2+^ and Hg^2+^ ions (Figure 4c,d).

Still related to those results, we measured the relative average fluorescence of Hg^2+^ ions, including changes in the intensity (Table S7). The fluorescence intensity was almost unchanged, indicating that the addition of CH_3_NH_3_PbBr_3_ QDs does not increase Hg^2+^ ion selectivity because the generation of Hg(OH)_2_ in the aqueous solution results in a red shift of the absorption wavelength and a decrease in fluorescence intensity (Figure S30).^53^ In addition, the average relative fluorescence intensity of Cu^2+^ ions was analyzed (Table S8), and the addition of CH_3_NH_3_PbBr_3_ QDs increased the selectivity of Cu^2+^ by 1.4 times.

### Fluorescence Quenching Mechanisms by Copper Ion

**Figure 5:**
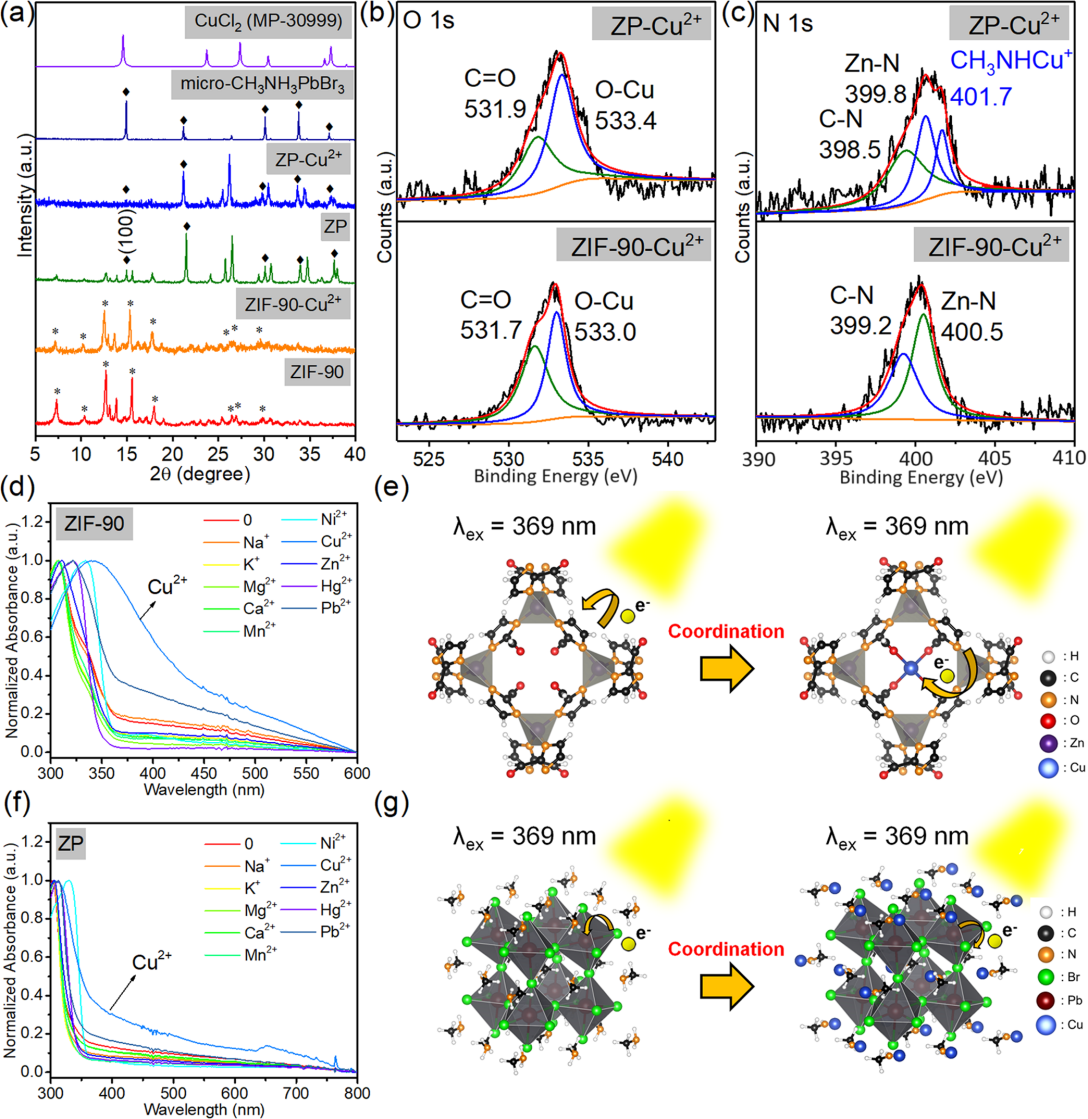
(a) XRD analysis of the structural changes of ZIF-90 and ZP composites with CuCl_2_ addition. XPS analysis of ZIF-90 and ZP composite bond energy changes of the (b) O 1s and (c) C 1s spectra. (d) Absorption spectra of ZIF-90 with various metal ion standard solutions and (e) the fluorescence quenching mechanism of ZIF-90 and Cu^2+^ ion coordination (e^–^ refers to electron from ICA that transferred during UV light irradiation). (f) Absorption spectra of ZP composite material with various metal ion standard solutions and (g) the fluorescence quenching mechanism of ZP and Cu^2+^ ion coordination (e^–^ refers to an electron from Br that transferred during UV light irradiation).

The conversion of CH_3_NH_3_PbBr_3_ to CH_3_NHCuPbBr_3_ may describe the metal ion selectivity in Figure 4d. We can see that the fluorescence quenching effect of ZP on Cu^2+^ ions in the metal selectivity test is significantly higher than that of ZIF-90. To analyze the quenching mechanism of Cu^2+^ ions for ZIF-90 and ZP composites, changes in the crystal structure were observed through XRD (Figure 5a).

The preparation for this observation was as follows. Standard CuCl_2_ aqueous solution was added to the two materials, and the mixture was reacted for half an hour. The powder obtained through centrifugation had no residual CuCl_2_ crystals. The crystal structure of the materials was examined. The result clarifies that adding Cu^2+^ ions to ZIF-90 did not change its ionic structure.

In addition, ZP composites containing Cu^2+^ were analyzed. In general, CH_3_NH_3_PbBr_3_ oxidation will form CH_3_NH_3_Br and PbBr_2_, respectively, so if CH_3_NH_3_PbBr_3_ is oxidized, the PbBr_2_ crystal diffraction peak will be observed. However, observing the reaction results of ZP and CuCl_2_ aqueous solution, it can be observed that the crystal structure of CH_3_NH_3_PbBr_3_ is consistent with that before the reaction without other redundant impurity phases. The (100) crystal plane of CH_3_NH_3_PbBr_3_ QDs changed, indicating a change in the A-site cation CH_3_NH_3_^+^.^23,54^

Further, bonding changes were analyzed through XPS to analyze the role of Cu^2+^ ions. The full XPS spectra of ZIF-90-Cu^2+^ and ZP-Cu^2+^ and details regarding their signals, such as C 1s, Cu 2p, Zn 2p, Pb 4f, and Br 3d, are presented in Figures S31–S33. When Cu^2+^ ions were added to ZIF-90 (Figure 5b), a 533.0 eV O–Cu bonding position appeared in ZIF-90, indicating that the ZIF-90 formaldehyde group provides a coordination bond for Cu^2+^. We also examined changes caused by the addition of Cu^2+^ ions to ZP composites. The O–Cu bond was observed in ZP composites. In addition, the A-site cation was converted from CH_3_NH_3_^+^ to 401.7 eV CH_3_NHCu^+^ (Figure 5c), and the energy spectrum analysis of Cu 2p revealed that it was a divalent Cu^2+^ ion (Figure S32b).^55^

A structural model was used to determine the change in the bonding energy between ZIF-90 and ZP composites. For ZIF-90, the kinetic diameter of Cu^2+^ (2.14 Å) had a high matching degree with the four aldehyde groups of the four-membered ring structure of ZIF-90, and this structure tended to coordinate with Cu^2+^ ions (Figure S34). By contrast, in ZF composites, the reaction of CH_3_NH_3_PbBr_3_ with Cu^2+^ resulted in the formation of CH_3_NH_2_. Subsequently, H^+^ and Cu^2+^ ions replaced each other to transform into CH_3_NHCu^+^, thus resulting in the formation of the perovskite structure of CH_3_NHCuPbBr_3_ (Figure S35).^21−23,28^

Analysis of optical properties after the addition of Cu^2+^ ions indicated that the absorption ranges of ZIF-90 and Cu^2+^ ions red-shifted to 400 nm (Figure 5d). When a 369 nm excitation light source irradiated ZIF-90, the electrons of the ligand HOMO transferred to the d9 half-spin orbital domain of Cu^2+^ ions (Figure 5e), resulting in the red-shifting of the ZIF-90 ligand to an absorption wavelength of 400 nm that corresponded to fluorescence quenching (as Figure 4c). On the other hand, CH_3_NHCu^+^ absorption was observed at 650 nm (Figure S36) after adding Cu^2+^ ions to the ZP composite apart from the red-shifting of ZIF-90 (Figure 5f). When CH_3_NH_3_PbBr_3_ was converted to CH_3_NHCuPbBr_3_, Br electrons in the perovskite octahedron structure were transferred to Cu^2+^ (Figure 5g), resulting in fluorescence quenching (Figure 4d).^55^

### Ion Diffusion Kinetics

To prove that ZIF-90 can coordinate Cu^2+^ ions in an aqueous solution and that ZP can give higher selectivity than ZIF-90 as well as to analyze the Cu^2+^ ion sensing range of ZIF-90 and ZP composites, we used different concentrations of CuCl_2_ aqueous solution to determine the fluorescence sensing of ZIF-90 and ZP composites prepared using different ligand-to-Zn^2+^ mole ratios (Figures S37 and S38) at each maximum emission wavelength (490 nm for ZIF-90 and 515 nm for ZP composite). Changes in the relative fluorescence intensity of ZIF-90 at 490 nm and those of ZP composites at 515 nm were compared after fitting by a linear eq (Figures S39 and S40).

The fluorescence quenching curve of the coordination between ZIF-90 and divalent copper ions confirms that the ZIF-90 and Cu^2+^ ion coordination mechanism follows the Langmuir model adsorption type. When the number of Cu^2+^ ions was small (Figure S41a), the ZIF-90 crystal coordinated with Cu^2+^ ions through the surface of the four-membered ring structure of ZIF-90, resulting in a gradual decrease in the fluorescence quenching constant. When the concentration of CuCl_2_ aqueous solution was gradually increased (Figure S41b), Cu^2+^ ions diffused into the six-membered ring structure and mesoporous structure of ZIF-90. The four-membered ring structure inside the crystal was coordinated with Cu^2+^ ions, resulting in a rapid increase in the fluorescence quenching constant of ZIF-90.

When the concentration of CuCl_2_ aqueous solution was considerably high, active sites on the ZIF-90 crystal surface and the internal four-membered ring structure coordinating with Cu^2+^ ions were saturated. Excess Cu^2+^ ions in water dynamically balanced the production of CuCl_2_, and the remaining excess CuCl_2_ produced weak fluorescence (Figure S42). At a low concentration of CuCl_2_ (Figure S41c), Cu^2+^ ions coordinated with the four-membered ring structure on the ZIF-90 crystal surface. At a high concentration of CuCl_2_ (Figure S41d), in ZP composites, CH_3_NH_3_PbBr_3_ QDs occupied the ZIF-90 crystal stack. Therefore, Cu^2+^ ions can diffuse into the crystal through the six-membered ring structure. These findings indicate that ZIF-90 and CH_3_NH_3_PbBr_3_ QDs react with Cu^2+^ ions, resulting in the fluorescence quenching of both ZIF-90 and CH_3_NH_3_PbBr_3_ QDs.^57,58^

By examining the diffusion kinetics of Cu^2+^ ions, we determined that the relative intensities of the maximum emission wavelengths of ZIF-90 at 490 nm and those of ZP composites at 515 nm, linearly fitted with the Stern–Volmer equation, presented below^12,13,22,23,26,28,56^
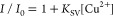
where *I*_0_ is the blank sample intensity, *I* is the sensed sample intensity, and *K*_SV_ is the fluorescence quenching constant.

**Figure 6:**
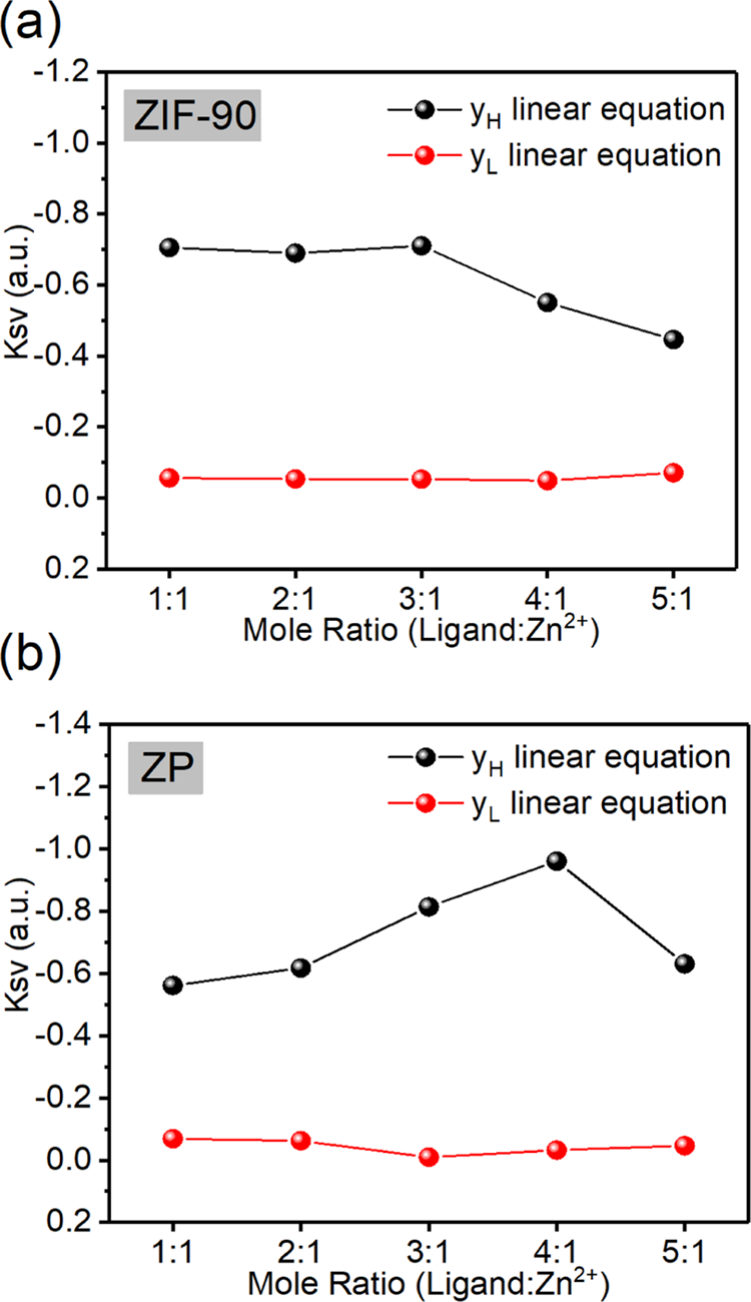
(a) Fluorescence quenching constants of ZIF-90 with different ligand-to-Zn^2+^ mole ratios fitted on the linear equations of *y*_H_ and *y*_L_. (b) Fluorescence quenching constants of ZP composites with different ligand-to-Zn^2+^ mole ratios fitted on the linear equations of *y*_H_ and *y*_L_.

To comply with the linear relationship, two linear distributions were defined. The concentration of the CuCl_2_ aqueous solution was 1 × 10^–2^ to 1 × 10^–3^ M, and its linear equation was referred to as *y*_H_. The lower concentration of CuCl_2_ aqueous solution ranged from 1 × 10^–3^ to 1 × 10^–7^ M, and its linear equation was referred to as *y*_L_. The *y*_H_ values of the ZIF-90 and ZP composites prepared using different ligand-to-Zn^2+^ mole ratios were compared with the fluorescence quenching constant of *y*_L_ (Tables S9 and S10). Fluorescence quenching constant *y*_L_ exerted a weak effect, while the fluorescence quenching constant y_H_ was much stronger. The ZIF-90 quenching constant decreased as the ligand-to-Zn^2+^ mole ratio gradually increased (Figure 6a). By contrast, the fluorescence quenching constant of the ZP composite reached the highest value when the ligand-to-Zn^2+^ mole ratio was 4:1 (Figure 6b).

### Defects and Fluorescence Quenching Constants

**Figure 7:**
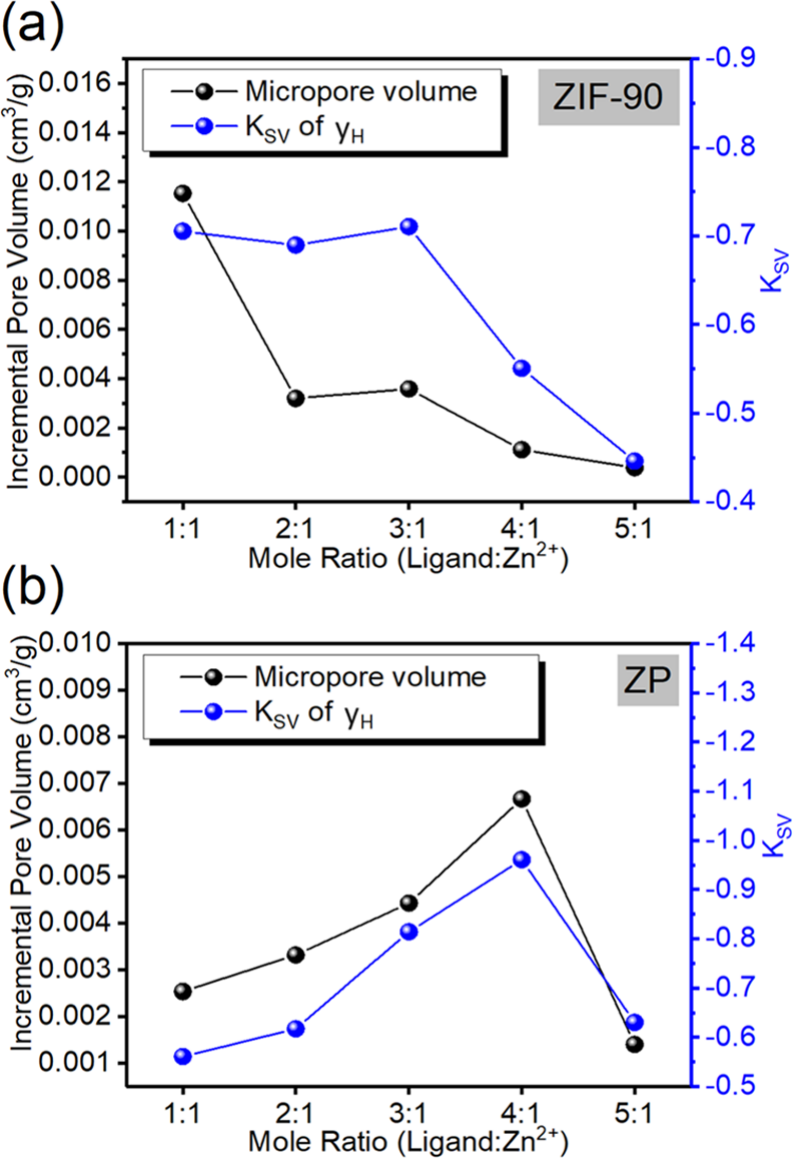
Relationship between micropore volume and fluorescence quenching constant for (a) ZIF-90 and (b) ZP composites.

The higher the ligand-to-Zn^2+^ mole ratio of ZIF-90 was, the lower the fluorescence quenching constant was. Because the kinetic diameter of micropores is close to that of Cu^2+^ ions, the intracrystalline diffusion of Cu^2+^ ions inside the ZIF-90 crystal exerted a crucial effect on the fluorescence quenching constant.^5,8,9,12^ When the ZIF-90 ligand defect produced a partial vacancy (Figure S43), the diffusion rate of Cu^2+^ ions in the ZIF-90 crystal increased. The ZIF-90 1:1 micropore had the highest pore volume (Figure 7a) because of the defect and thus had the highest fluorescence quenching constant.^8,37,58^ When CH_3_NH_3_PbBr_3_ QDs were added and occupied gaps in the crystal stack, excess CH_3_NH_3_PbBr_3_ QDs reduced the diffusion rate of Cu^2+^ ions (Figure 7b).

When the number of excess CH_3_NH_3_PbBr_3_ QDs was decreased (e.g., ZP 4:1 composite), ZIF-90 and CH_3_NH_3_PbBr_3_ QDs exhibited the highest quenching constant, which was considered the best formulation for Cu^2+^ ions sensing. Thus, the fluorescence quenching constant of ZP was higher than that of ZIF-90. However, the fluorescence quenching constant of the ZP 5:1 composite material decreased significantly because excess micro-CH_3_NH_3_PbBr_3_ caused the agglomeration of ZIF-L crystals. This caused Cu^2+^ ions to preferentially react with micro-CH_3_NH_3_PbBr_3_ in aqueous solution, resulting in a considerable decrease in the fluorescence quenching constant.

To calculate the limit of detection (LOD) of ZIF-90 and ZP composites toward Cu^2+^, the following equation was used^12,59^
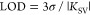
where σ is the standard deviation of 10 blank samples and |*K*_SV_| is the absolute value of the fluorescence quenching constant. The standard deviation of the solution was 3.02765. For sensing CuCl_2_, the minimum limit of detection of ZIF-90 1:1 was 1.28 × 10^–2^ M, and that of ZP 4:1 was 0.95 × 10^–2^ M (Table 1). The findings indicate that CH_3_NH_3_PbBr_3_ QDs can sense a lower-concentration CuCl_2_ solution.

**Table 1:** Lowest Sensing Limit of CuCl_2_ Aqueous Solution Obtained with ZIF-90 and ZP Composites

mole ratio of ZIF-90 (ligand/Zn^2+^)	1:1	2:1	3:1	4:1	5:1
LOD (×10^–2^ M)	1.28	1.31	1.28	1.66	2.04
mole ratio of ZP (ligand/Zn^2+^)	1:1	2:1	3:1	4:1	5:1
LOD (×10^–2^ M)	1.62	1.47	1.11	0.95	1.44

Moreover, we compared some relative works that utilized the combination of MOF and perovskite for fluorescence sensing (Table S11). The comparison shows that our works’ material using a novel approach, defect engineering, can give a good result for specific Cu^2+^ ion sensing. This result cannot be separated from the role of ZIF-90s selective site we used for chelating Cu^2+^ (which other materials do not have) and defect engineering for loading more perovskite and interacting with Cu^2+^ and increasing our materials’ selectivity and sensitivity toward Cu^2+^ ions.

## Conclusions

In summary, we successfully prepared the ZIF-90/CH_3_NH_3_PbBr_3_ composite by providing defect sites in ZIF-90 crystals for loading perovskite QDs. This combination was proposed to improve ZIF-90 fluorescence sensing ability and, simultaneously, study its effect in the field of defect engineering. The findings of this study indicate that the higher the ligand-to-Zn^2+^ mole ratio in ZIF-90 synthesis, the lower the number of ligand defects and the more complete the crystal stacking is. However, the number of ZIF-L as the ZIF-90 impurity phase was relatively large when the ligand ratio was higher. We then added CH_3_NH_3_PbBr_3_ QDs to prepare the ZP composites. BET and HRTEM findings indicated the presence of CH_3_NH_3_PbBr_3_ QDs was mainly in the crystal stacking gap and stabled them during application in water. The Cu^2+^ ion selectivity of the ZIF-90 and ZP composites was examined through XRD and XPS, which indicated that ZIF-90 could derive O–Cu bonds while in ZP, CH_3_NH_3_PbBr_3_ transformed into CH_3_NHCuPbBr_3_, resulting in the quenching of fluorescence. The fluorescence quenching constant of ZIF-90 prepared using different ligand-to-Zn^2+^ mole ratios was obtained by fitting the Stern–Volmer equation by the ion diffusion kinetics approach and then compared with that of the ZP composites. The fluorescence quenching constant positively correlated with ZIF-90 crystal defects. After the addition of CH_3_NH_3_PbBr_3_ QDs, the fluorescence quenching constant was −0.9606. The lowest sensing limit for CuCl_2_ aqueous solutions was 0.95 × 10^–2^ M. Thus, this method is suitable for developing fluorescence sensing of Cu^2+^ ion in water and gives more insight into the relationship between MOFs defect engineering and fluorescence sensing.

## Supporting Information Available

Detailed information about the structure illustration; detailed SEM-EDS images and tables of the prepared materials; complete the materials N_2_ adsorption–desorption graphics; detailed images and tables of HRTEM results; complete XPS spectra of the prepared materials; UV–vis spectra of the materials followed by their calculation of the lowest excited state energy; complete fluorescence emissions spectra of the materials under different pH values, metal solutions, and CuCl_2_ concentrations, followed by their fitted linear equation data; and comparison with other published works and references (PDF)
