# Supplementary material for: Defect Engineering Strategy for Superior Integration of Metal–Organic Framework and Halide Perovskite as a Fluorescence Sensing Material
Source: ACS Appl Mater Interfaces. 2024 Apr 23;16(24):31023–35. doi: 10.1021/acsami.4c00770 (PMC11194771; doi:10.1021/acsami.4c00770)
Supplement: Supplementary file 1 — am4c00770_si_001.pdf [file am4c00770_si_001.pdf]

Supporting Information

Defect Engineering Strategy for Superior Integration of Metal-Organic Framework and Halide Perovskite as Fluorescence Sensing Material

Zhun-Xian Lai [a,†], Andi Magattang Gafur Muchlis [a,†], Ramadhass Keerthika Devi [a,d], Chen-Lung Chiang [a], Yi-Ting Syu [a], Yi-Ting Tsai [a], Cuo-Chi Lee [c], and Chun Che Lin [a,b,*]

[a] Institute of Organic and Polymeric Materials, National Taipei University of Technology, Taipei, 106334, Taiwan
[b] Research and Development Center for Smart Textile Technology, National Taipei University of Technology, Taipei, 106334, Taiwan
[c] Department of Agricultural Science and Technology, Ministry of Agriculture, Taipei, 100, Taiwan
[d] Department of Biomedical Science, Chang Gung University, Taoyuan City, 33302, Taiwan

† These authors contributed equally.
* Corresponding author, E-mail: cclin0530@mail.ntut.edu.tw (C. C. Lin)

Tables:

Table S1. Element percentage of ZIF-90.

Element	Weight (%)		Atomic (%)	
Calculation method	Ideal	Measurement	Ideal	Measurement
C	35.31	42.33	47.06	56.42
N	20.58	21.62	23.53	24.71
O	11.76	13.29	11.76	13.29
Zn	24.03	22.76	5.88	5.57
Totals	91.68	100.00	88.23	100.00

Table S2. Element percentage of ZP composites.

Element	Weight (%)	Atomic (%)
Calculation method	Measurement	Measurement
C	37.87	53.19
N	20.85	25.11
O	14.90	15.70
Zn	19.90	5.13
Br	2.62	0.55
Pb	3.86	0.31
Totals	100.00	100.00

Table S3. Energy dispersive element distribution of ZIF-90, micro-$CH_3NH_3PbBr_3$, and ZP composites determined through HRTEM.

Material	Element	Weight (%)	Atomic (%)
ZIF-90	Zn	100.00	100.00
Micro-$CH_3NH_3PbBr_3$	Br	43.10	66.26
	Pb	56.90	33.74
ZP composites	Zn	0.28	0.72
	Br	12.95	27.69
	Pb	86.78	71.59

Table S4. Average fluorescence quenching intensity of ZIF-90 dispersed in different acid–base solutions.

pH	1	2	3	4	5	6	7
Average F_{490} I/I_0 (%)	50.7	90.5	95.5	98.6	97.0	98.1	100.0
pH	8	9	10	11	12	13	—
Average F_{490} I/I_0 (%)	99.2	100.1	102.1	99.3	90.5	36.3	—

Table S5. Average fluorescence quenching intensity of ZP composites dispersed in different acid–base solutions.

pH	1	2	3	4	5	6	7
Average F_{515} I/I_0 (%)	19.3	30.1	80.9	90.76	94.0	91.0	100.0
pH	8	9	10	11	12	13	—
Average F_{515} I/I_0 (%)	97.8	97.5	88.2	71.7	61.5	18.1	—

Table S6. pH values of different chloride salt aqueous solutions.

Metal salts	NaCl	KCl	$MgCl_2$	$CaCl_2$	$MnCl_2$
pH	4.53	5.03	7.30	5.66	5.38
Metal salts	$NiCl_2$	$CuCl_2$	$ZnCl_2$	$HgCl_2$	$PbCl_2$
pH	7.93	3.06	6.58	3.91	3.63

Table S7. Selectivity of ZIF-90 and ZP composites dispersed in 1×10^{-2} M aqueous $HgCl_2$ solution.

Hg^{2+} selectivity			
ZIF-90		ZP composites	
Ligand:Zn^{2+}	F_{515} I/I_0	Ligand:Zn^{2+}	F_{515} I/I_0
1 : 1	0.701	1 : 1	0.670
2 : 1	0.739	2 : 1	0.780
3 : 1	0.850	3 : 1	0.792
4 : 1	0.685	4 : 1	0.780
5 : 1	0.822	5 : 1	0.719
Average	0.759	Average	0.746

Table S8. Selectivity of ZIF-90 and ZP composites dispersed in 1 × 10^{-2} M aqueous $CuCl_2$ solution.

Cu^{2+} selectivity			
ZIF-90		ZP composites	
Ligand:Zn^{2+}	F_{490} I/I_0	Ligand:Zn^{2+}	F_{490} I/I_0
1 : 1	0.758	1 : 1	0.389
2 : 1	0.626	2 : 1	0.470
3 : 1	0.800	3 : 1	0.552
4 : 1	0.721	4 : 1	0.702
5 : 1	0.757	5 : 1	0.624
Average	0.732	Average	0.547

Table S9. Fitting data of the relative fluorescence intensity of ZIF-90 prepared using different ligand-to-Zn^{2+} mole ratios and concentrations of $CuCl_2$ aqueous solution.

$CuCl_2$ (M)	0.01 to 0.1		1 × 10^{-2} to 1 × 10^{-7}	
Mole Ratio (Ligand:Zn^{2+})	Y_H slope (K_{SV})	Fitted value (R)	Y_L slope (K_{SV})	Fitted value (R)
1:1	-0.70536	0.97896	-0.05612	0.88530
2:1	-0.68968	0.85663	-0.05340	0.99713
3:1	-0.71055	0.90090	-0.05267	0.98764
4:1	-0.55036	0.99483	-0.04868	0.95299
5:!	-0.44605	0.98668	-0.07073	0.99196

Table S10. Fitting data of the relative fluorescence intensity of ZP composites prepared using different ligand-to-Zn^{2+} mole ratios and concentrations of $CuCl_2$ aqueous solution.

$CuCl_2$ (M)	0.01 to 0.1		1 × 10^{-2} to 1 × 10^{-7}	
Mole Ratio (Ligand:Zn^{2+})	Y_H slope (K_{SV})	Fitted value (R)	Y_L slope (K_{SV})	Fitted value (R)
1:1	-0.56119	0.96039	-0.06904	0.96916
2:1	-0.61751	0.97534	-0.06223	0.91636
3:1	-0.81429	0.95427	-0.00947	0.73635
4:1	-0.96060	0.98454	-0.03223	0.88016
5:!	-0.63045	0.98283	-0.04641	0.92912

Table S11. Reference comparison of related research of MOFs' combination with perovskites for fluorescence detection or sensing.

Ref. No.	MOF	Perovskite	Method	Analyte	Detection range or LOD (M)
[1]	MOF-5	$CH_3NH_3PbBr_3$	Two-step in situ synthesis method	Al^{3+}, Bi^{3+}, Co^{2+}, Cu^{2+}, Fe^{3+}, and Cd^{2+}	Range Cu^{2+} = 2 to 20 × 10^{-4}
[2]	ZIF-8$Co_{5\%}$	$MAPbBr_3$	One pot synthesis	Fe^{3+}	LOD = 3.9 × 10^{-5}
[3]	ZIF-8	$MAPbBr_3$	One pot synthesis	ClO^{-}	LOD = 3.19 × 10^{-8}
[4]	In-ppda-1 and In-pdda-2	$MAPbBr_3$	The "ship in a bottle" method	Gossypol	LOD = 2.86 × 10^{-8}
[5]	ZIF-8 + Pb	$CH_3NH_3PbBr_3$	Pb-doping (two-step solution-based) method	Glutathione	LOD = 3.6 × 10^{-10}
[6]	MOF-5	$CsPbBr_3$	In situ synthesis (MOF-5 + Perovskite)	Cd^{2+}	LOD = 1.192 × 10^{-7}
[7]	ZIF-90	-	-	Cd^{2+}, Cu^{2+}, CrO_4^{2-}, and acetone	Range Cu^{2+} = 0.001 to 5 × 10^{-4}
This work	ZIF-90	$CH_3NH_3PbBr_3$	Defect engineering + two-step in situ synthesis method	Cu^{2+}	LOD = 0.95 × 10^{-2}

Figures:

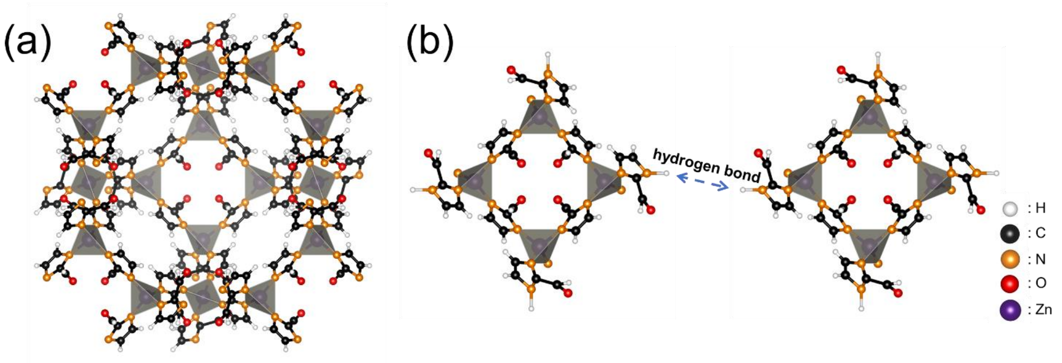

Figure S1. (a) ZIF-90 structure; (b) ZIF-L structure.

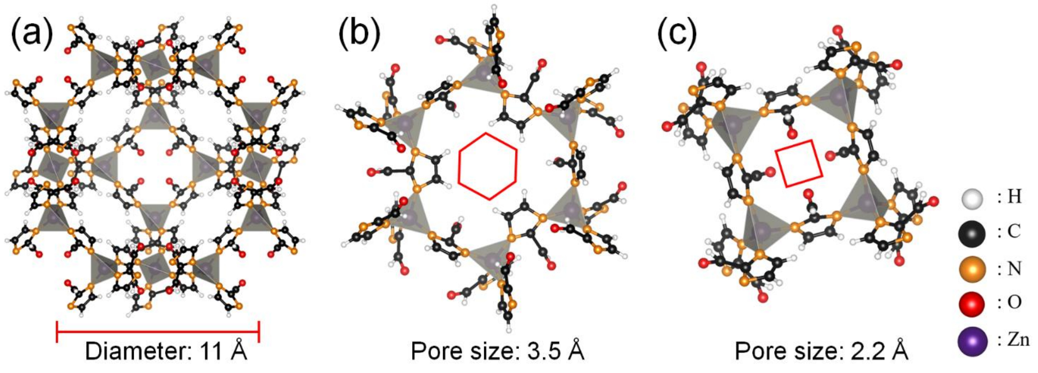

Figure S2. Crystal structure of ZIF-90 consists of the (a) cavity, (b) six-membered ring, and (c) four-membered ring.

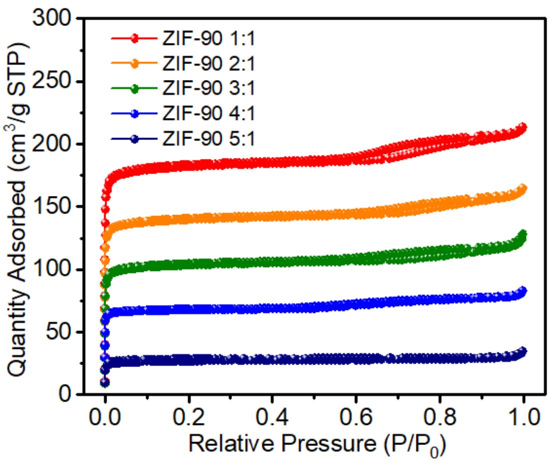

Figure S3. Nitrogen adsorption and desorption specific surface area diagrams of ZIF-90 prepared using different ligand-to-Zn^{2+} mole ratios.

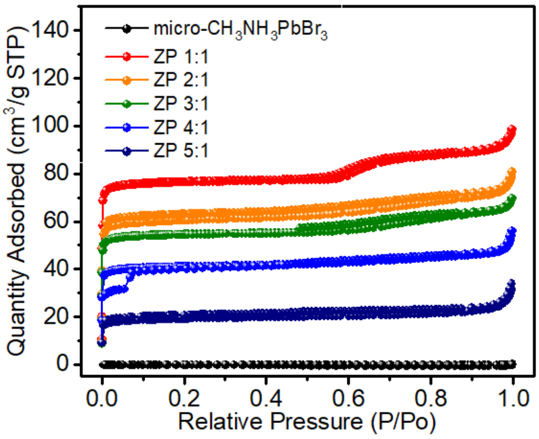

Figure S4. Nitrogen adsorption and desorption specific surface area diagrams of ZP composites prepared using different ligand-to-Zn^{2+} mole ratios.

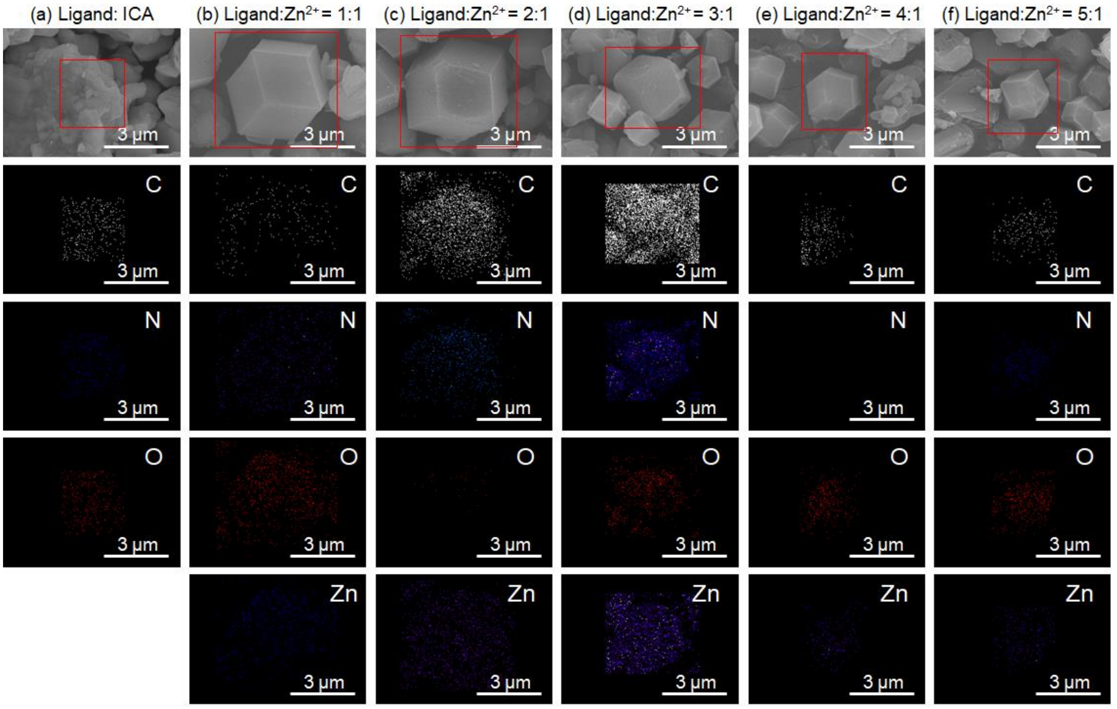

Figure S5. Elemental distribution of (a) ICA and ZIF-90 prepared using ligand-to-Zn^{2+} mole ratios of (b) 1:1, (c) 2:1, (d) 3:1, (e) 4:1, and (f) 5:1.

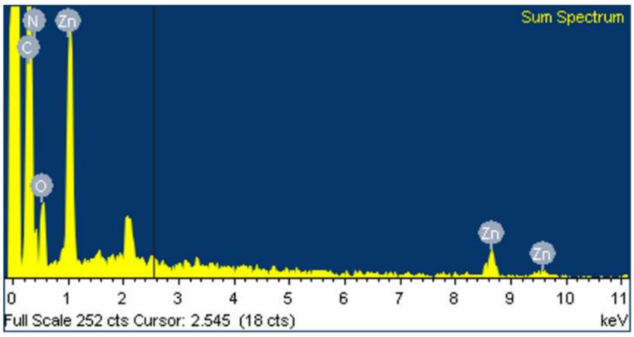

Figure S6. Energy dispersive distribution of ZIF-90.

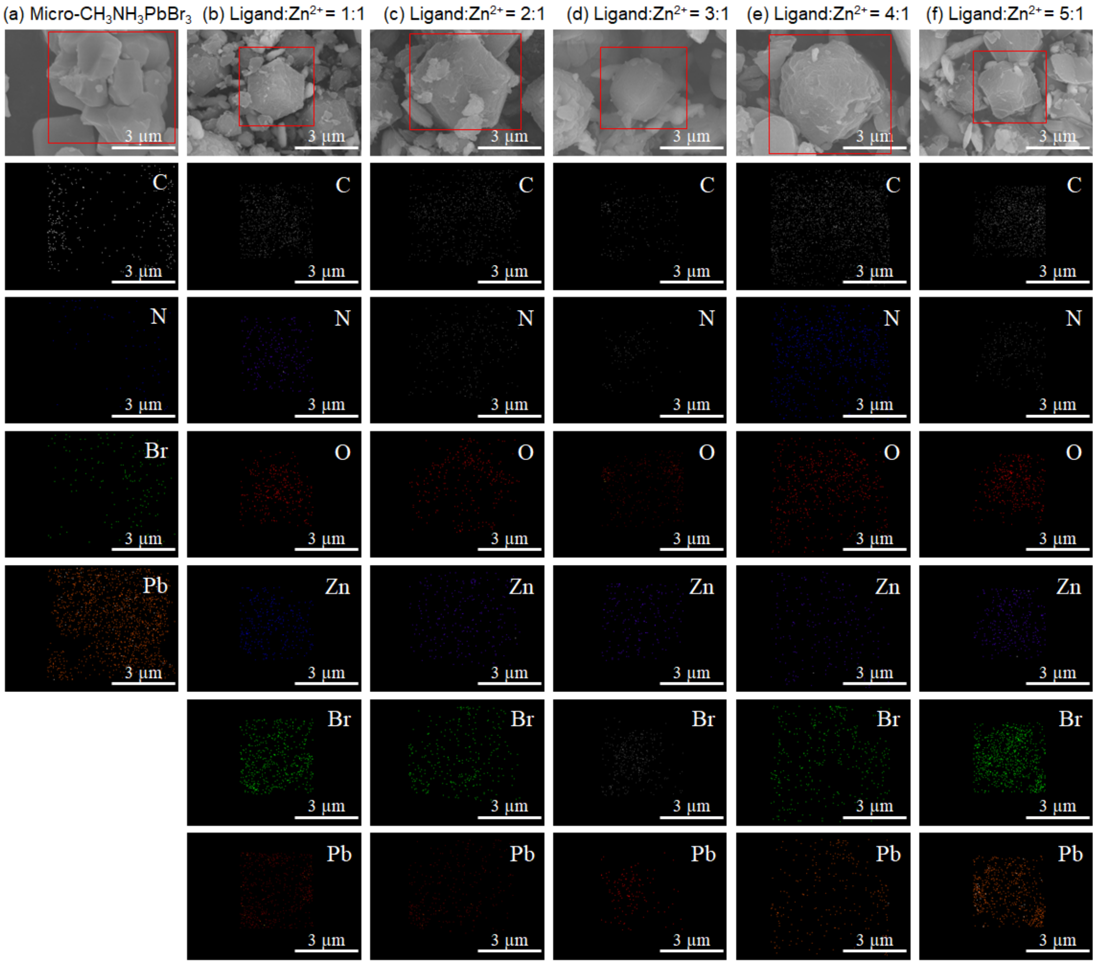

Figure S7. Elemental distribution of (a) micro-$CH_3NH_3PbBr_3$ and ZP composites prepared using ligand-to-Zn^{2+} mole ratios of (b) 1:1, (c) 2:1, (d) 3:1, (e) 4:1, and (f) 5:1.

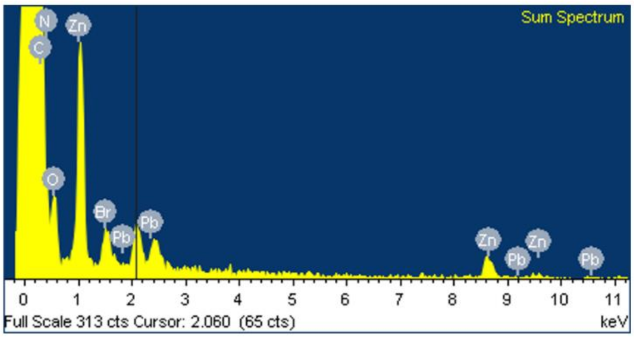

Figure S8. Energy dispersive distribution of ZP composites.

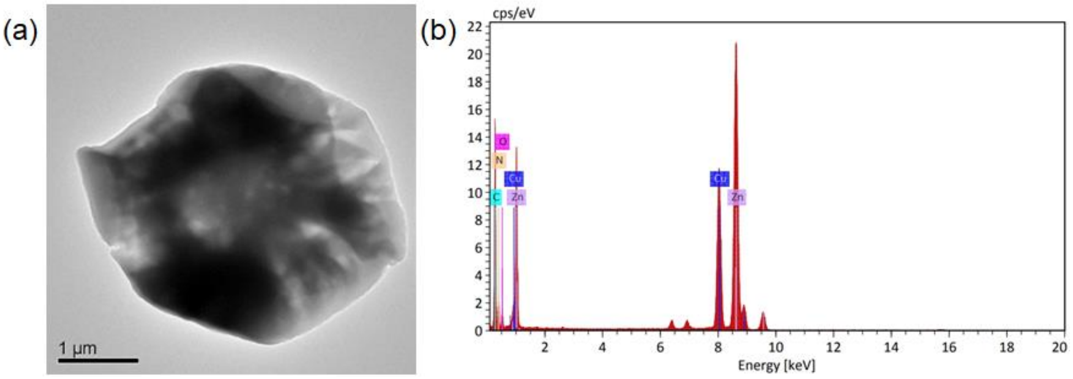

Figure S9. (a) HRTEM image of ZIF-90 and its (b) energy dispersive distribution spectrum.

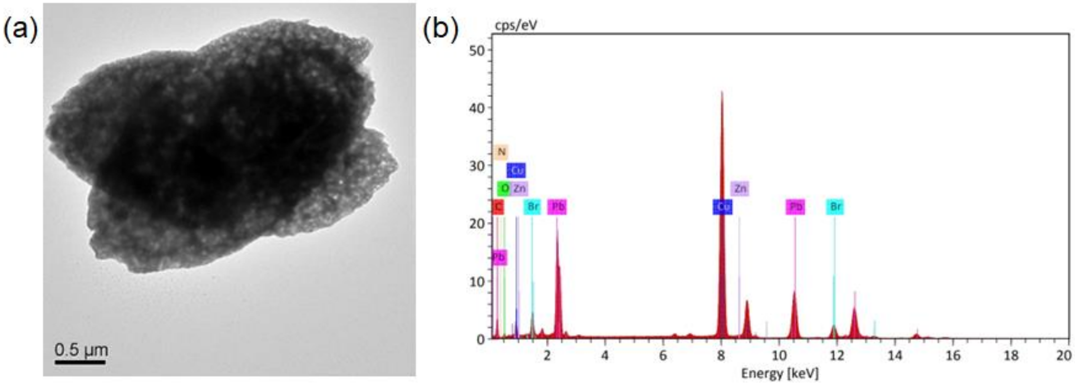

Figure S10. (a) HRTEM image of ZP composites and its (b) energy dispersive distribution spectrum.

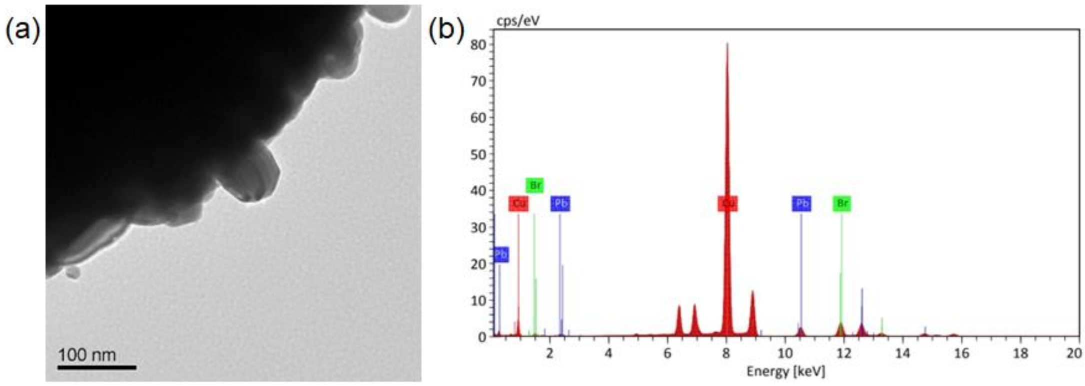

Figure S11. (a) HRTEM image of micro-$CH_3NH_3PbBr_3$ and its (b) energy dispersive distribution spectrum.

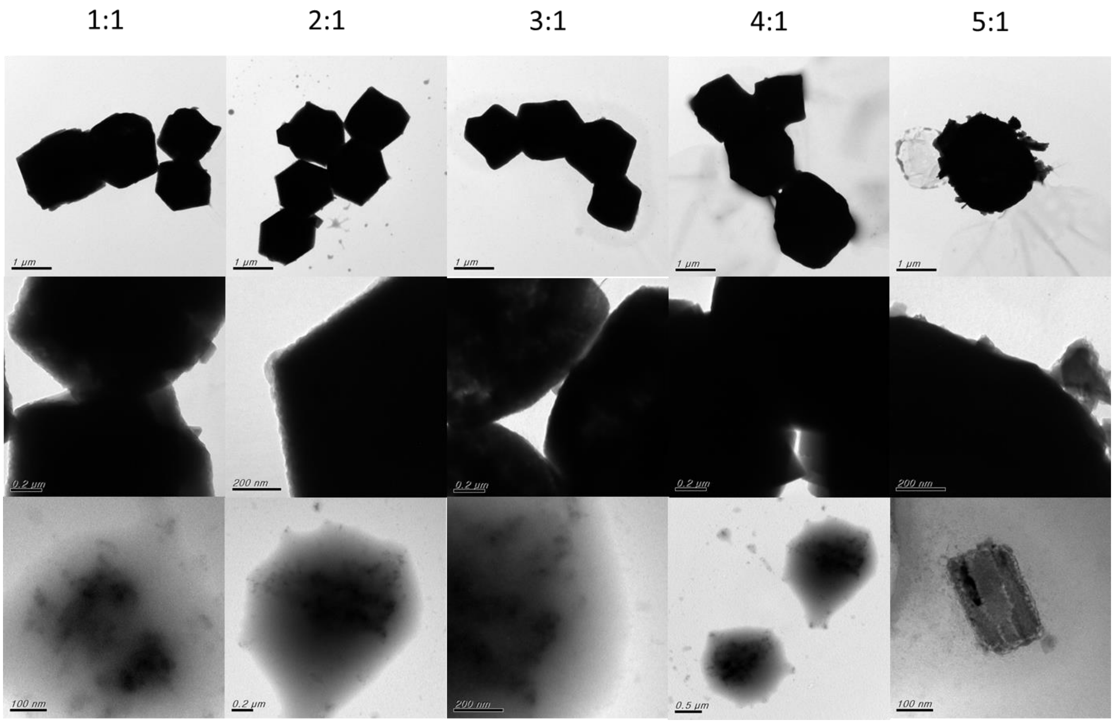

Figure S12. TEM images of ZIF-90 prepared using ligand-to-Zn^{2+} mole ratios 1:1, 2:1, 3:1, 4:1, and 5:1.

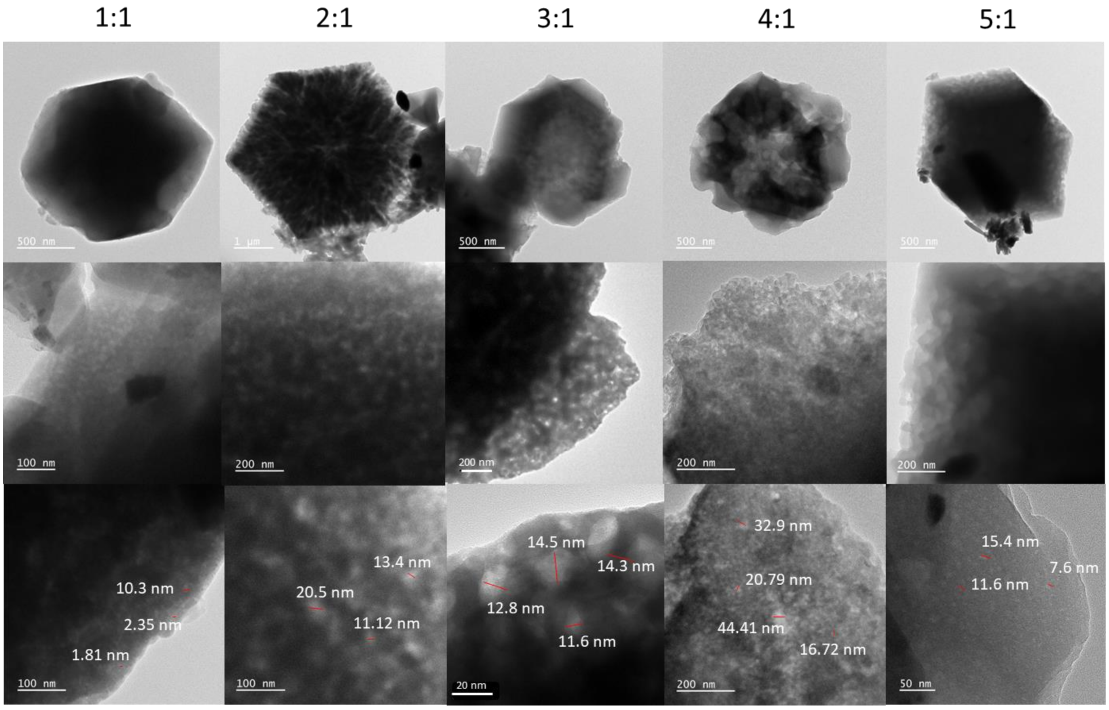

Figure S13. HR-TEM images of ZP composites with ligand-to-Zn^{2+} mole ratios of 1:1, 2:1, 3:1, 4:1, and 5:1.

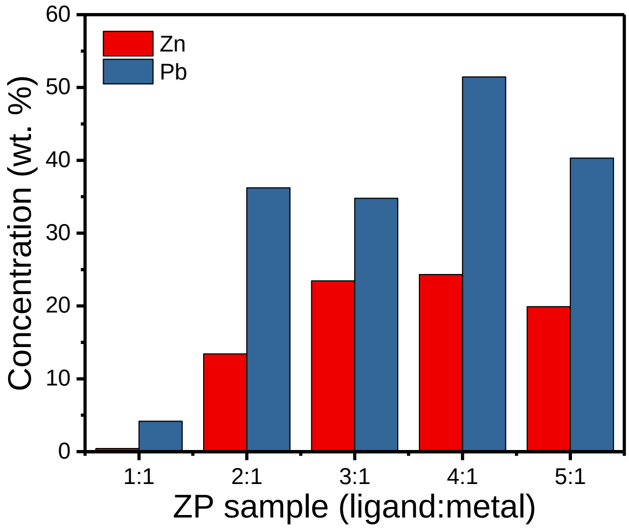

Figure S14. ICP-OES data to analyse Zn and Pb concentration in each sample of ZP composite.

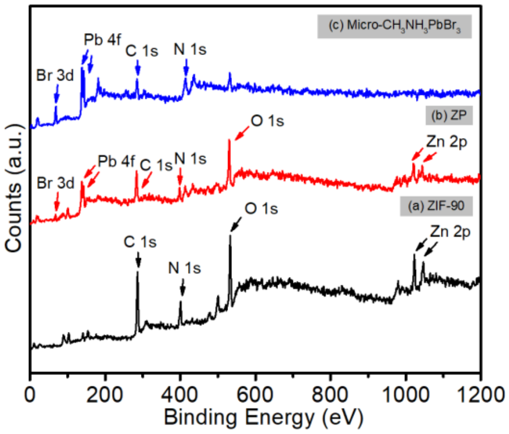

Figure S15. XPS full spectrum of (a) ZIF-90, (b) ZP composites, and (c) micro-$CH_3NH_3PbBr_3$.

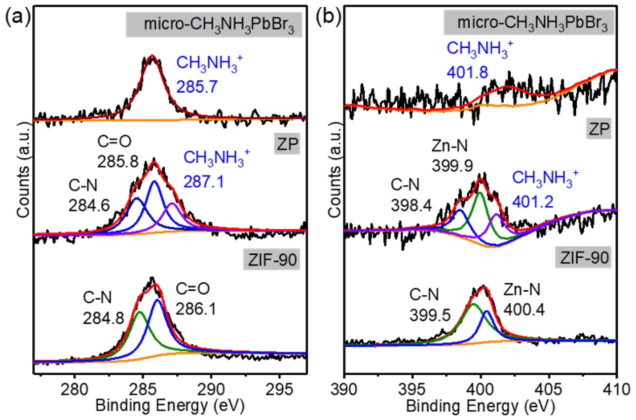

Figure S16. ZIF-90, ZP composites, and micro-$CH_3NH_3PbBr_3$ XPS element signal for (a) C 1s and (b) N 1s.

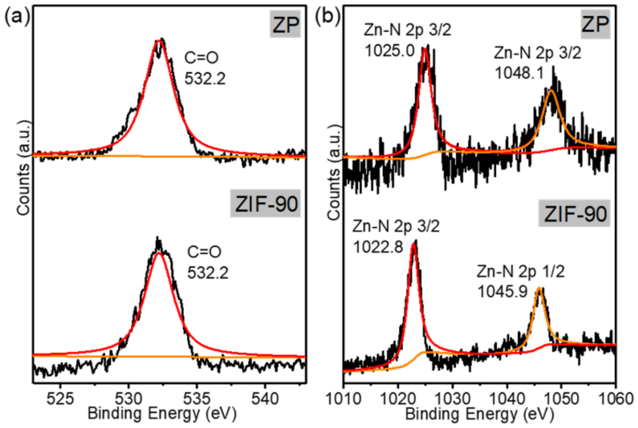

Figure S17. ZIF-90 and ZP composite XPS element signal for (a) O 1s and (b) Zn 2p.

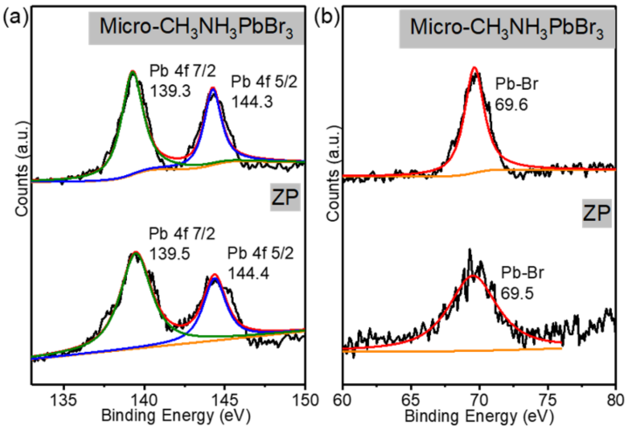

Figure S18. ZP composite and micro-$CH_3NH_3PbBr_3$ XPS element signal for (a) Pb 4f and (b) Br 3d.

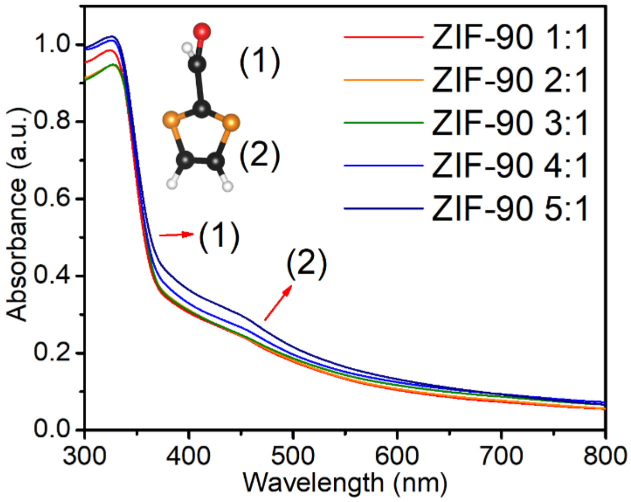

Figure S19. UV-vis absorption spectrum of ZIF-90 prepared using different ligand-to-Zn^{2+} mole ratios.

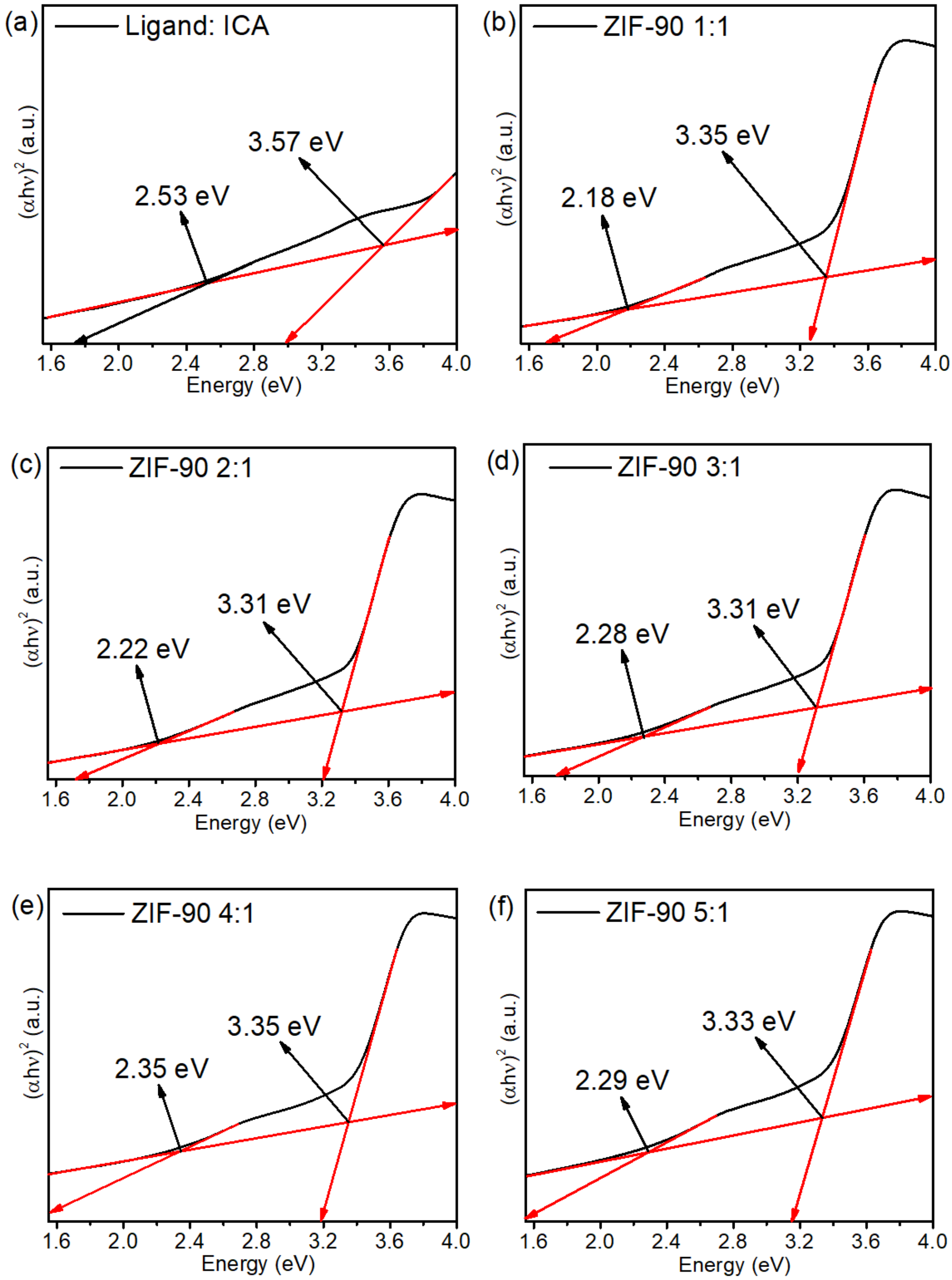

Figure S20. The lowest excited state energies of (a) ICA and ZIF-90 with different ligand-to-Zn^{2+} mole ratios of (b) 1:1, (c) 2:1, (d) 3:1, (e) 4:1, and (f) 5:1.

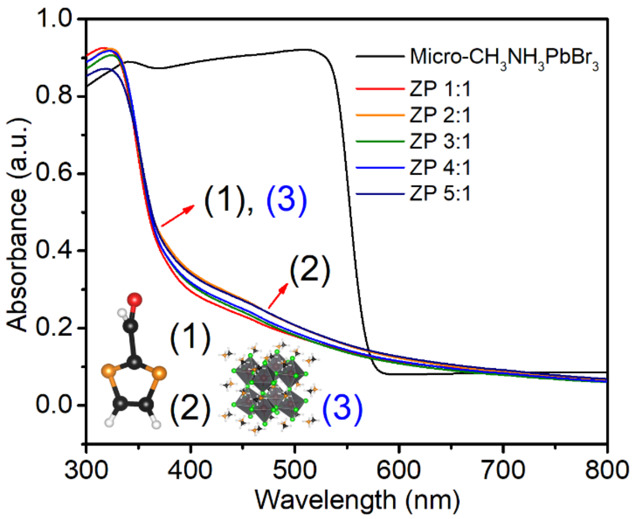

Figure S21. UV-Vis absorption spectrum of ZP prepared using different ligand-to-Zn^{2+} mole ratios and micro-$CH_3NH_3PbBr_3$.

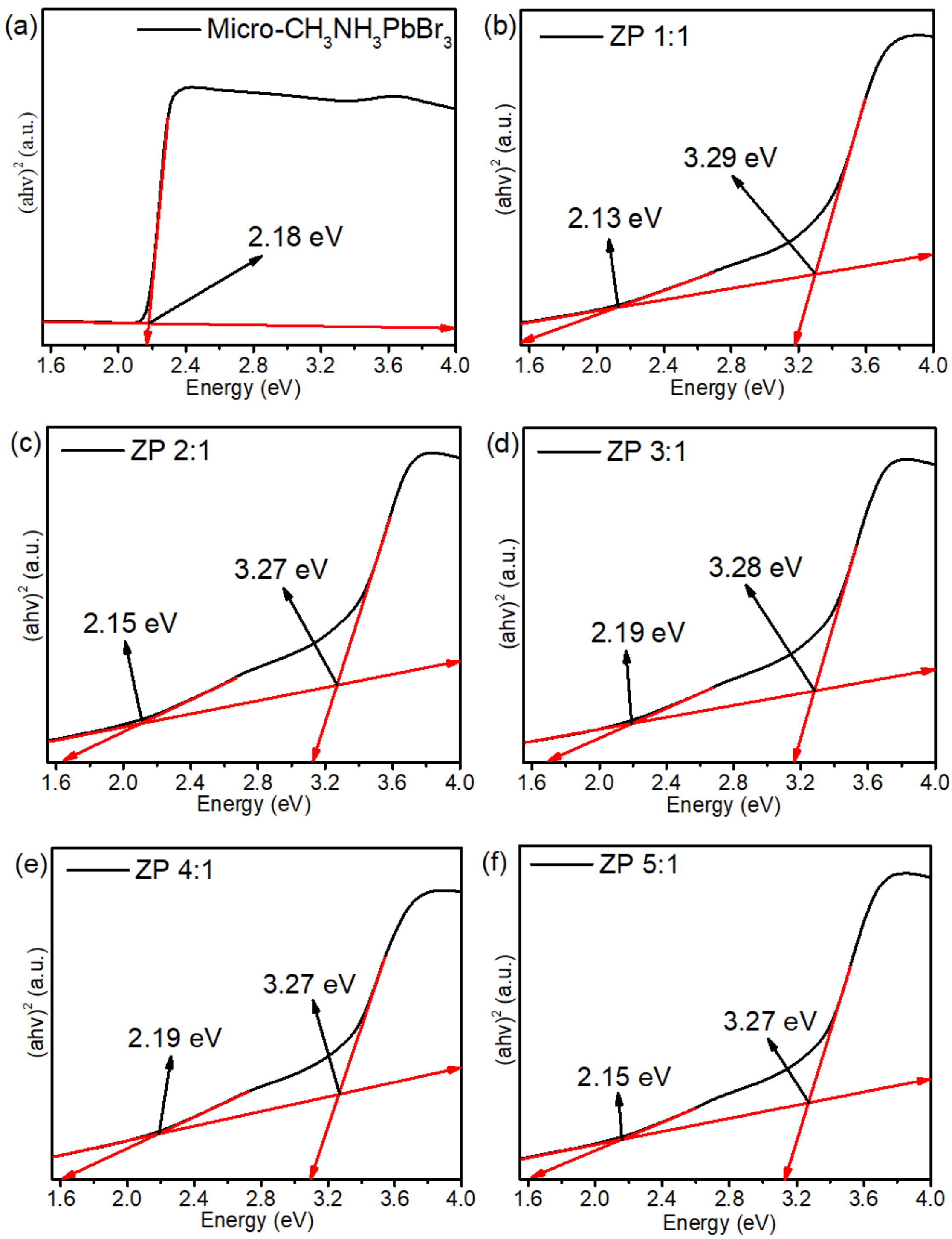

Figure S22. The lowest excited state energies of (a) micro-$CH_3NH_3PbBr_3$ and ZP composites prepared using ligand-to-Zn^{2+} mole ratios of (b) 1:1, (c) 2:1, (d) 3:1, (e) 4:1, and (f) 5:1.

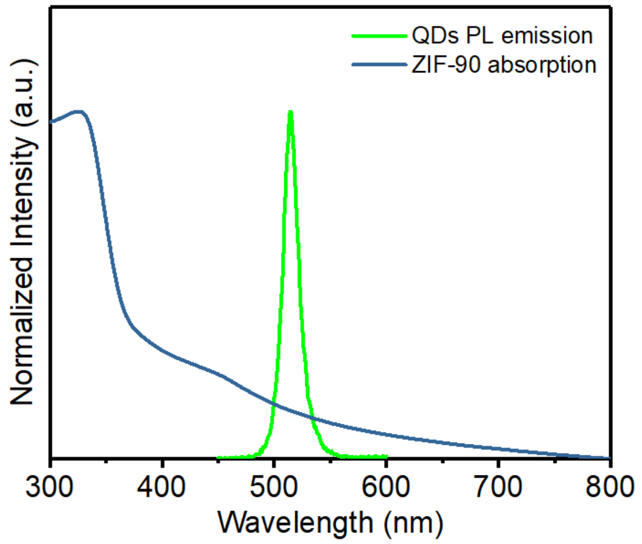

Figure S23. UV-Vis absorption spectrum of ZIF-90 compared to QDs fluorescence emission spectrum.

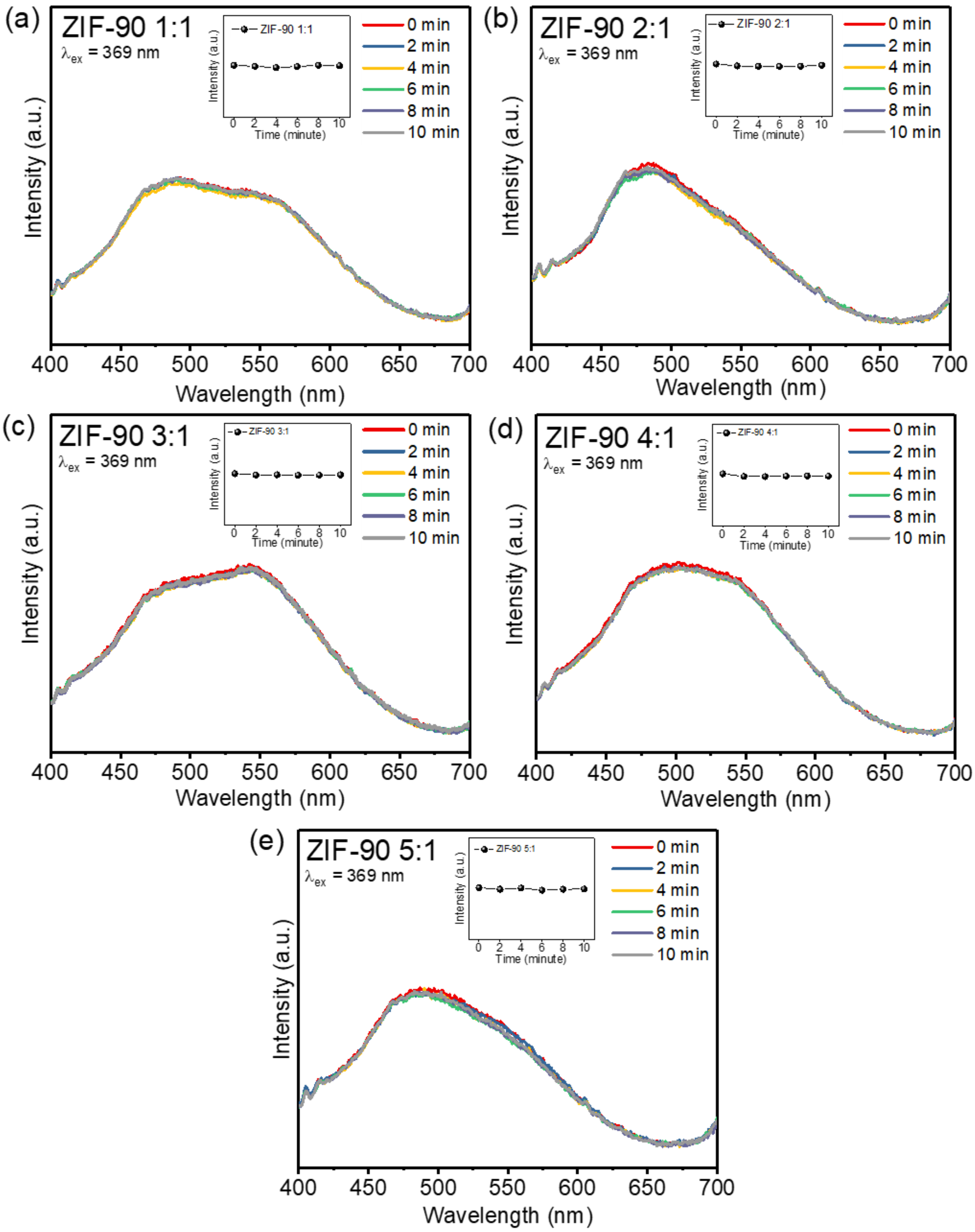

Figure S24. Fluorescence emission spectra of ZIF-90 with ligand-to-Zn^{2+} mole ratios of (a) 1:1, (b) 2:1, (c) 3:1, (d) 4:1, and (e) 5:1 in water for various times.

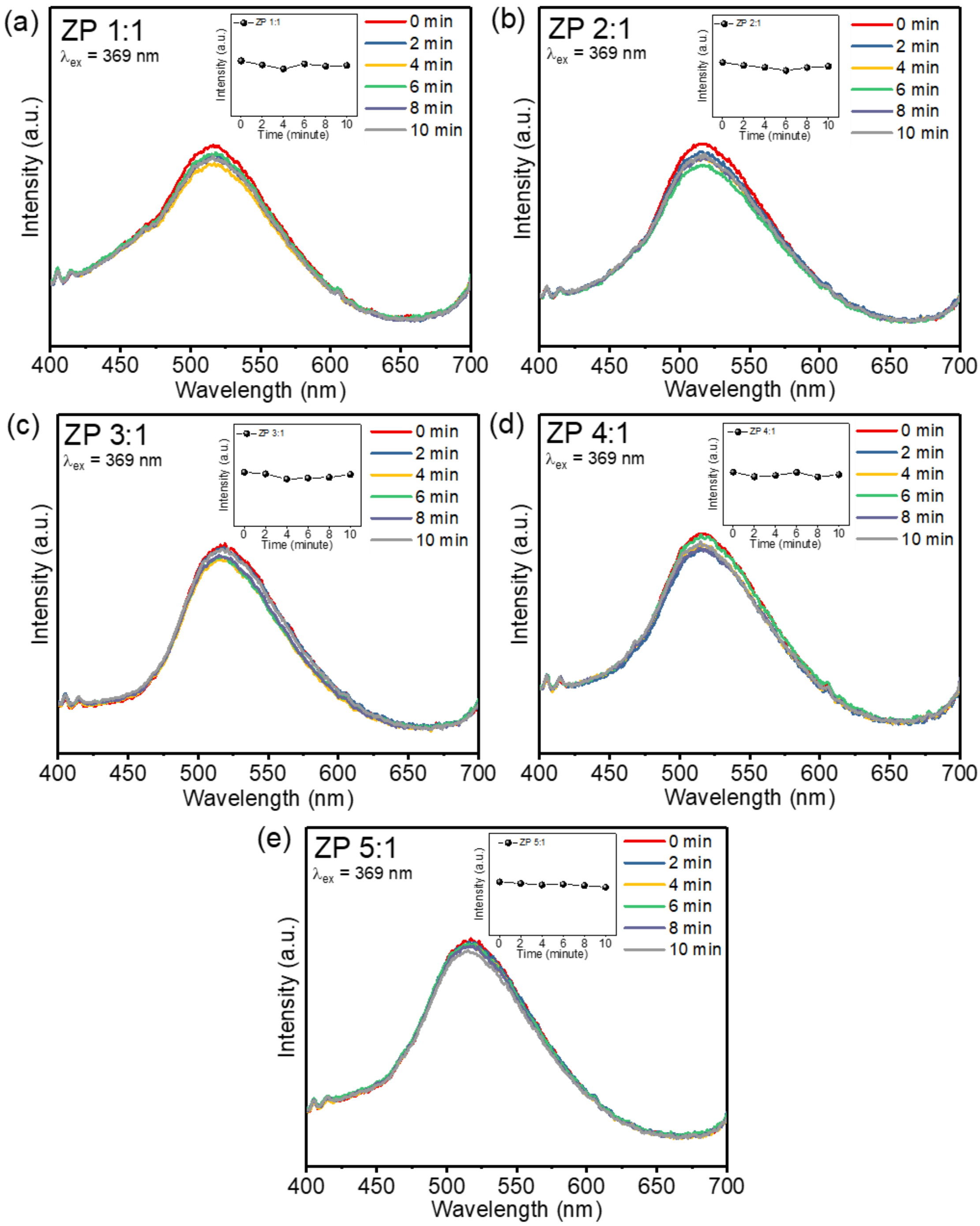

Figure S25. Fluorescence emission spectra of ZP composites with ligand-to-Zn^{2+} mole ratios of (a) 1:1, (b) 2:1, (c) 3:1, (d) 4:1, and (e) 5:1 in water for various times.

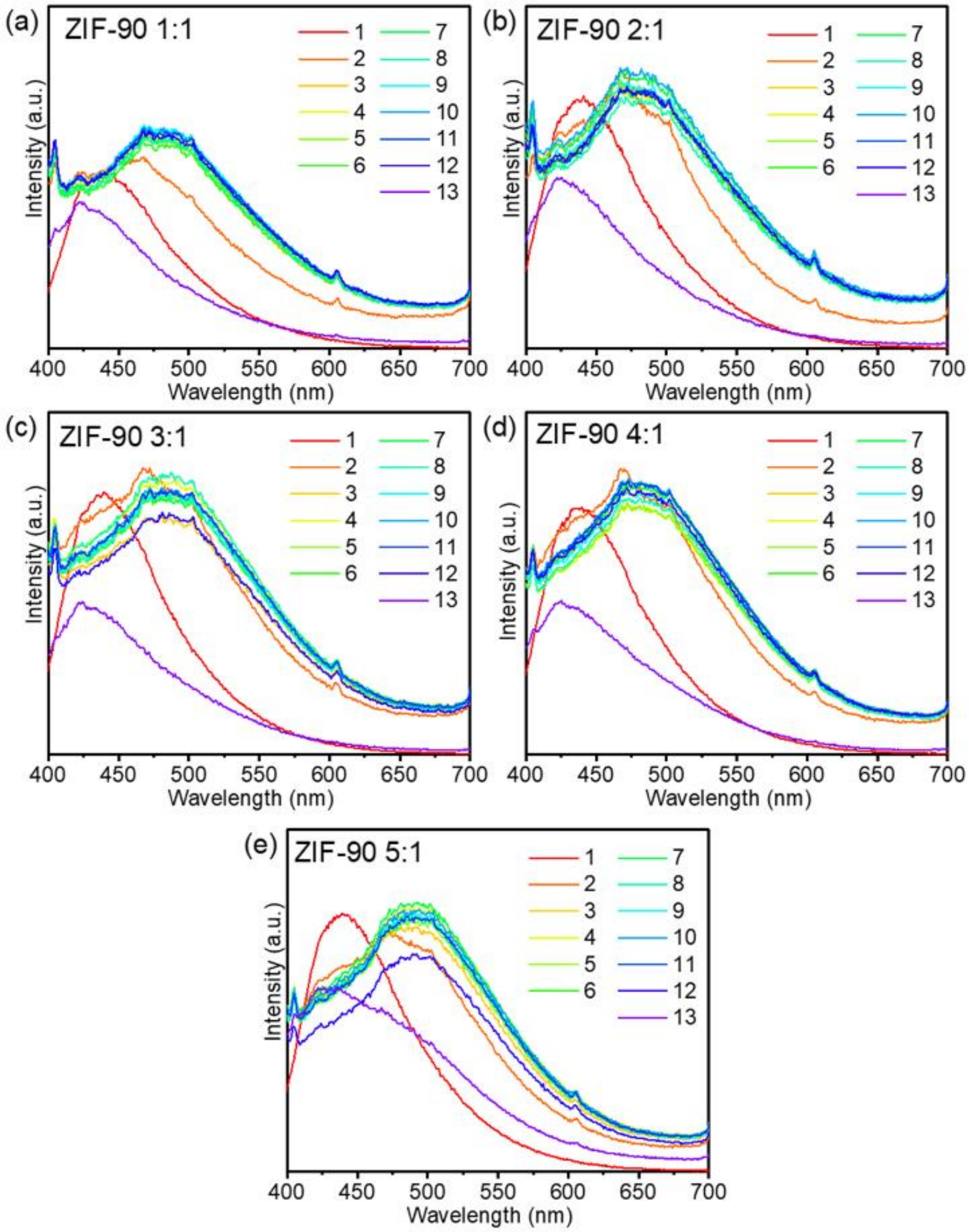

Figure S26. Fluorescence emission spectra of ZIF-90 in the acid–base tolerance range of ligand-to-Zn^{2+} mole ratios of (a) 1:1, (b) 2:1, (c) 3:1, (d) 4:1, and (e) 5:1.

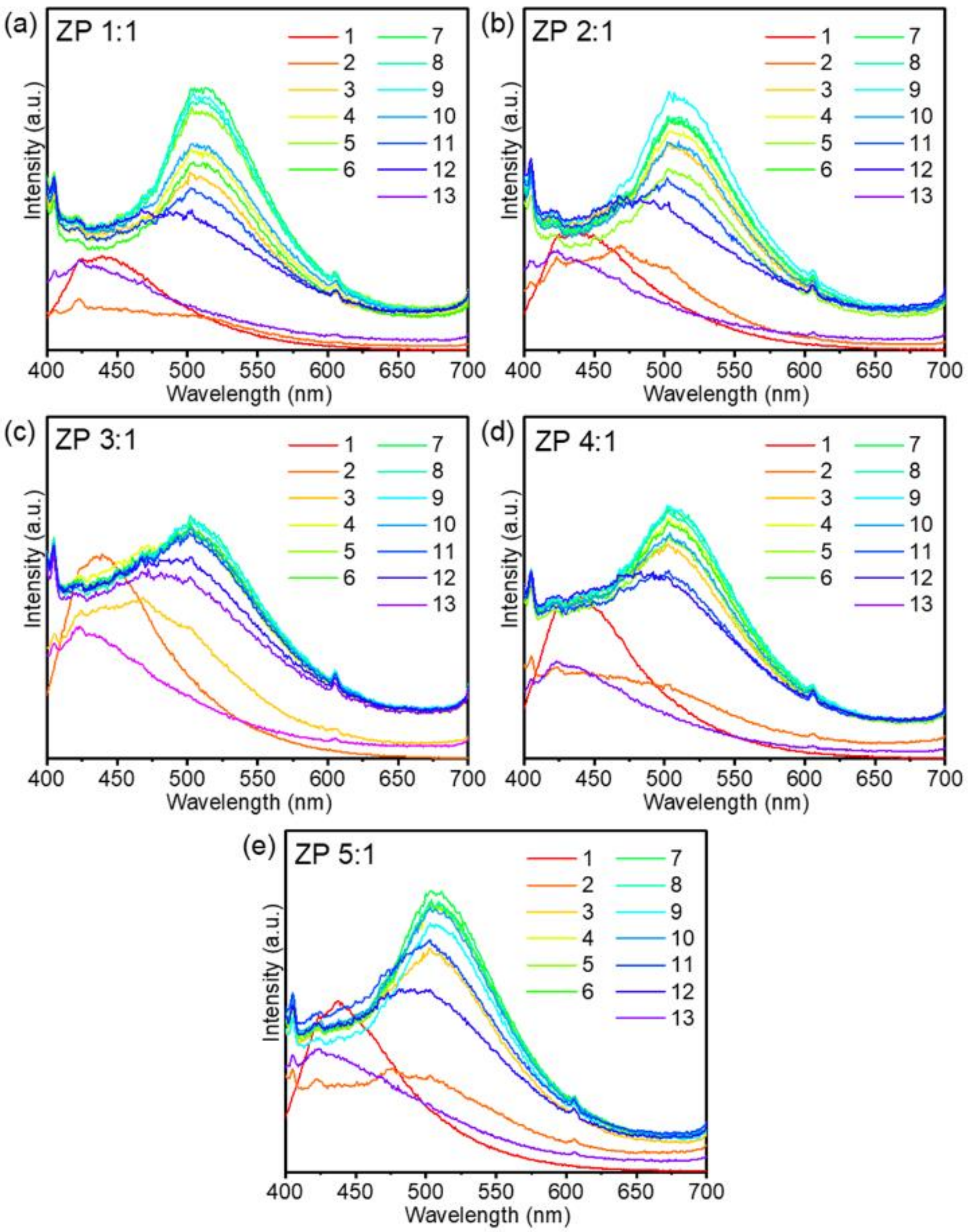

Figure S27. Fluorescence emission spectra of ZP composites in the acid−base tolerance range with ligand-to-Zn^{2+} mole ratios of (a) 1:1, (b) 2:1, (c) 3:1, (d) 4:1, and (e) 5:1.

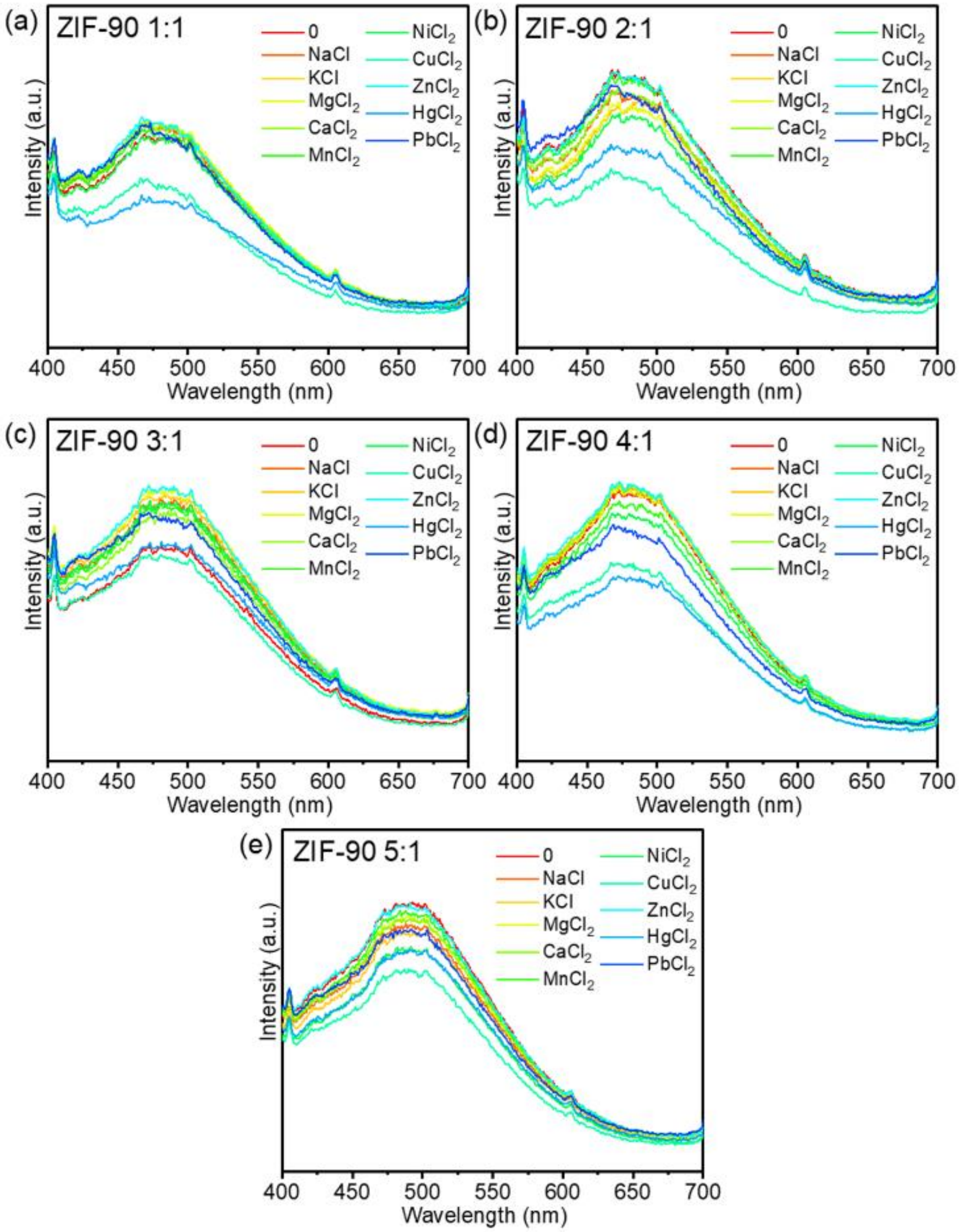

Figure S28. Fluorescence emission spectra of ZIF-90 with ligand-to-Zn^{2+} mole ratios of (a) 1:1, (b) 2:1, (c) 3:1, (d) 4:1, and (e) 5:1 in several types of metal solution.

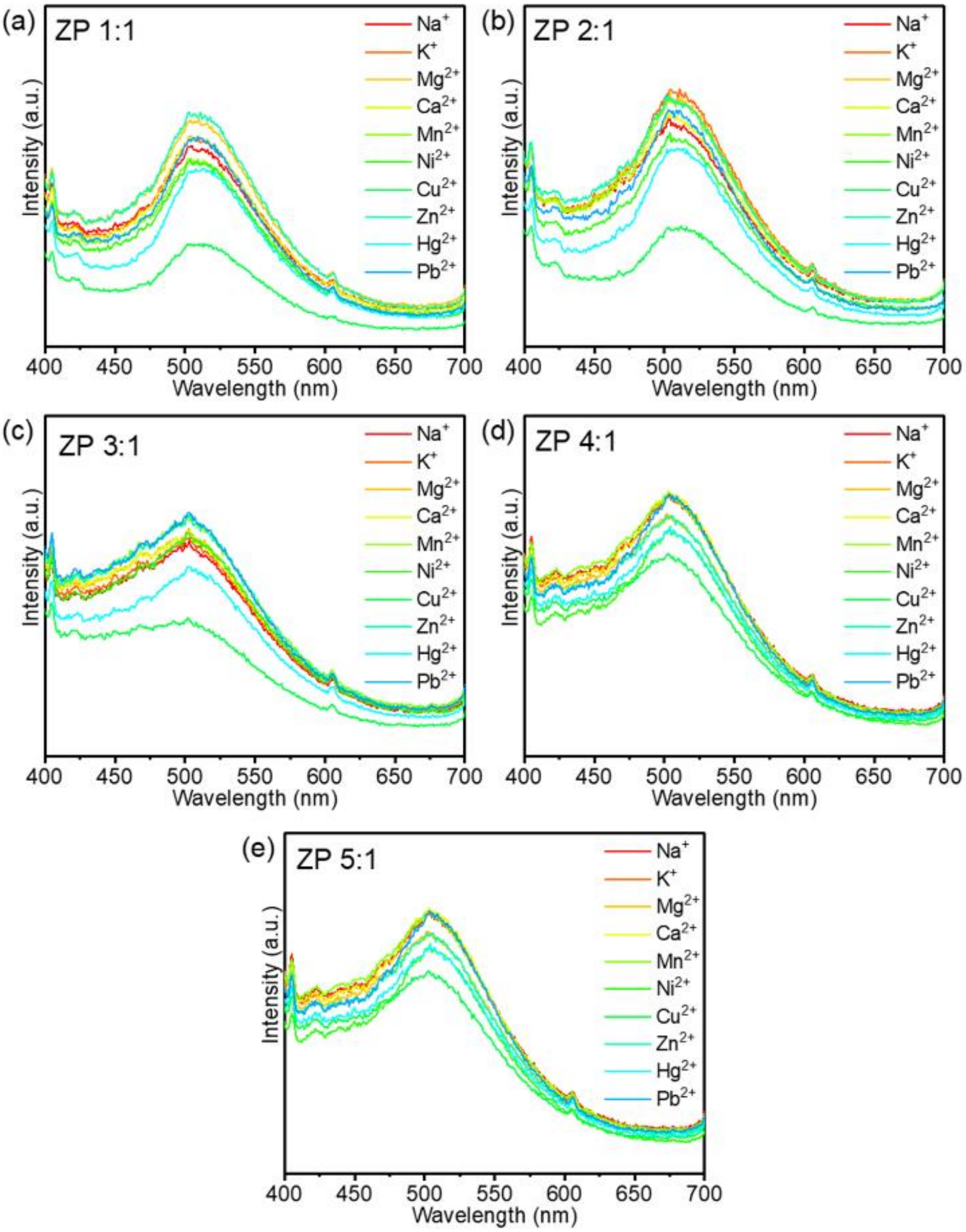

Figure S29. Fluorescence emission spectra of ZP composites prepared using ligand-to-Zn^{2+} mole ratios of (a) 1:1, (b) 2:1, (c) 3:1, (d) 4:1, and (e) 5:1 in several types of metal solution.

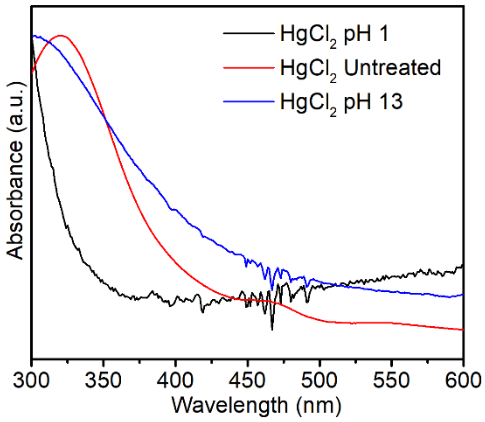

Figure S30. UV-Vis absorption spectrum of 1 × 10^{-2} M $HgCl_2$ added to different acid–base standard solutions.

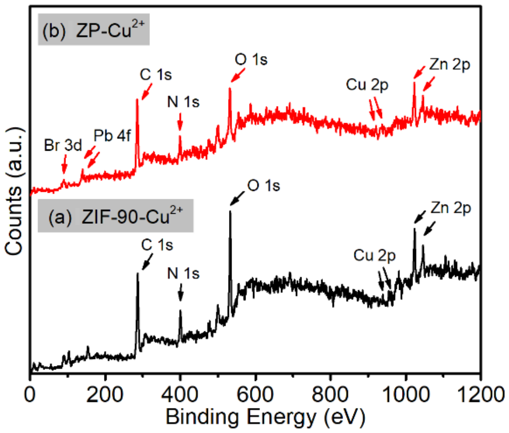

Figure S31. XPS full spectrum of (a) ZIF-90-Cu^{2+} and (b) ZP-Cu^{2+}.

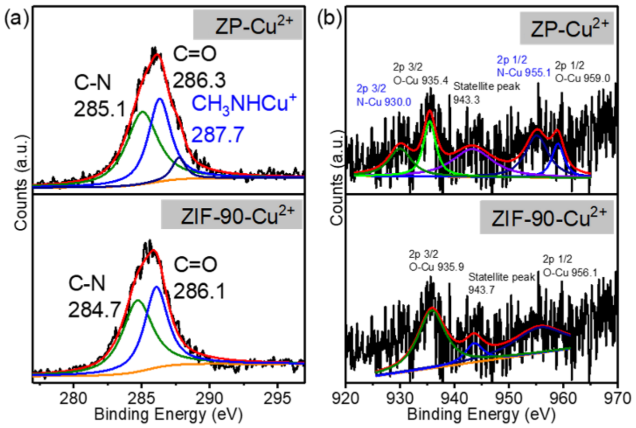

Figure S32. ZIF-90-Cu^{2+} and ZP-Cu^{2+} XPS element signal for (a) C 1s and (b) Cu 2p.

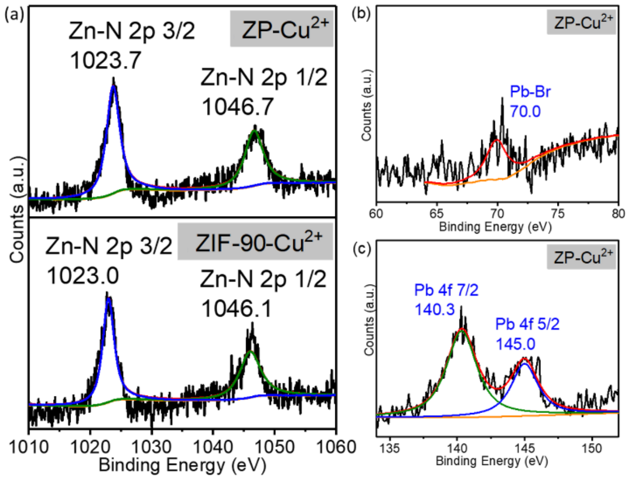

Figure S33. ZIF-90-Cu^{2+} and ZP-Cu^{2+} XPS element signal for (a) Zn 2p, (b) Br 3d, and (c) Pb 4f.

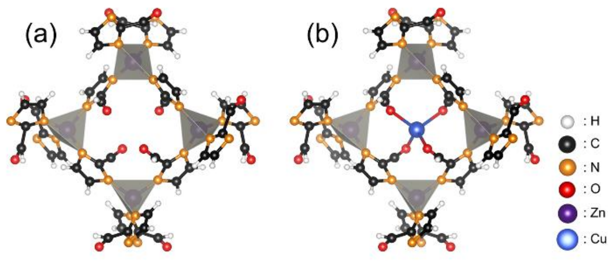

Figure S34. ZIF-90 four-membered ring structure after reacting with Cu^{2+} ions (a) before coordination and (b) after coordination.

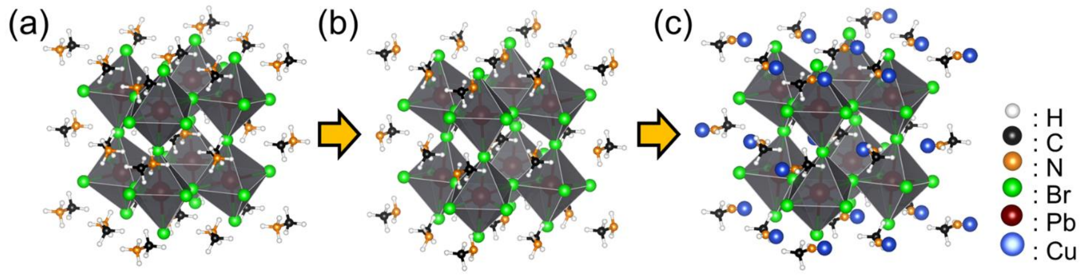

Figure S35. Structure of $CH_3NH_3PbBr_3$ after reacting with Cu^{2+} ions (a) $CH_3NH_3PbBr_3$, (b) CH_3NH_2-$PbBr_3^-$, and (c) $CH_3NHCuPbBr_3$.

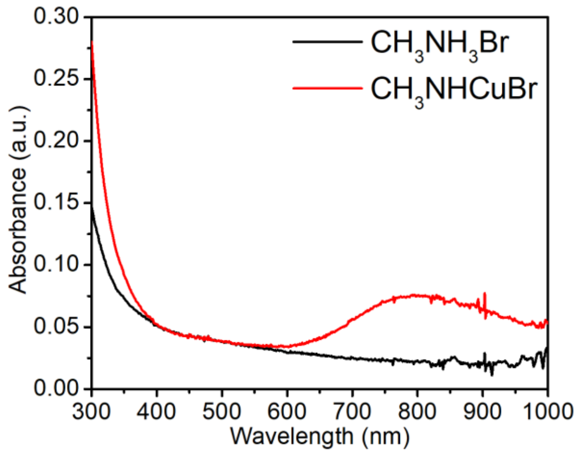

Figure S36. UV-Vis absorption spectrum of 0.2 mmol CH_3NH_3Br added to 1×10^{-2} M $CuCl_2$ standard solution.

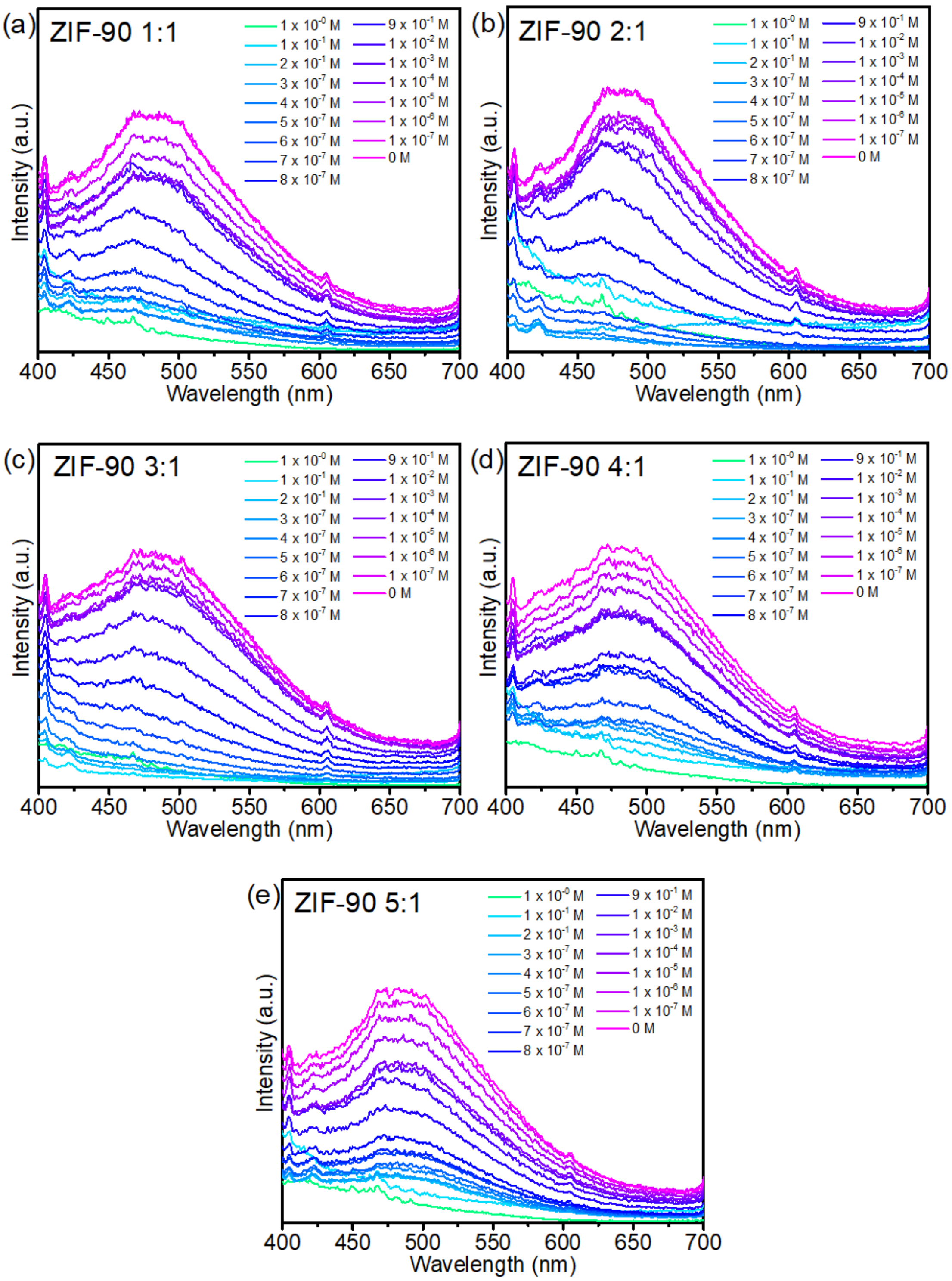

Figure S37. Fluorescence emission spectra of ZIF-90 prepared using different ligand-to-Zn^{2+} mole ratios in $CuCl_2$ aqueous solutions with molar concentrations of (a) 1:1, (b) 2:1, (c) 3:1, (d) 4:1, and (e) 5:1.

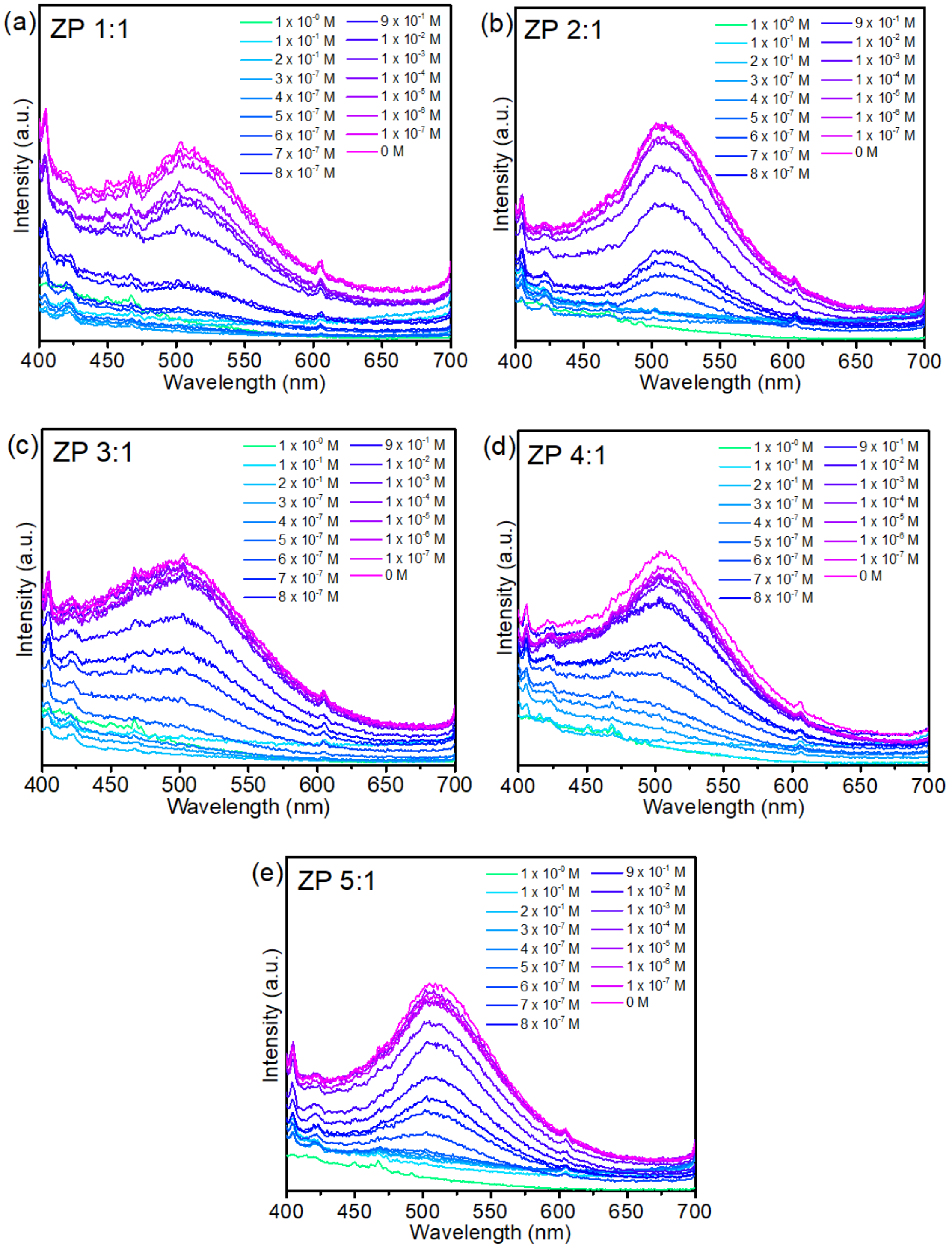

Figure S38. Fluorescence emission spectra of ZP composites prepared using different ligand-to-Zn^{2+} mole ratios in $CuCl_2$ aqueous solutions with molar concentrations of (a) 1:1, (b) 2:1, (c) 3:1, (d) 4:1, and (e) 5:1.

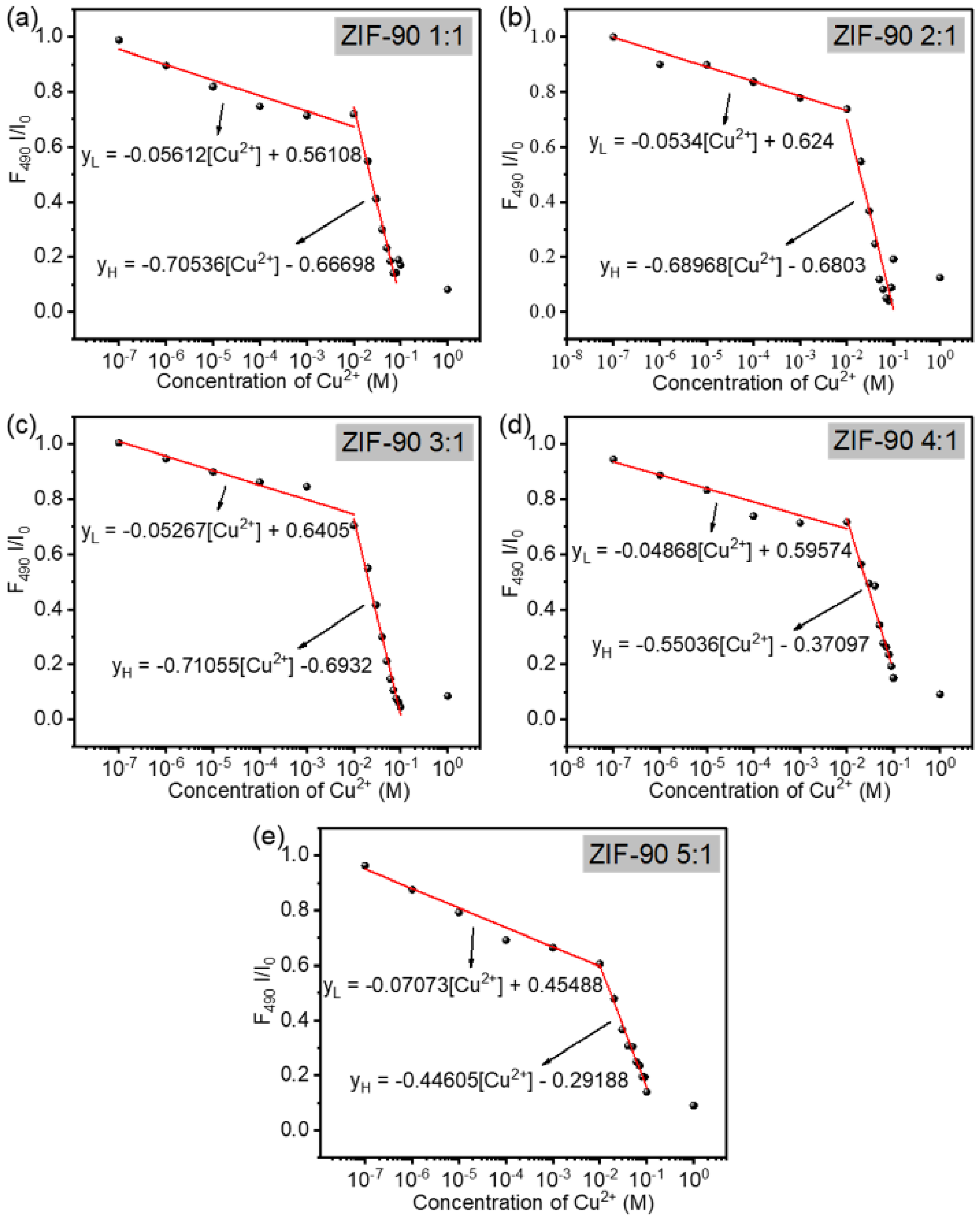

Figure S39. Relative fluorescence intensity of ZIF-90 prepared using different ligand-to-Zn^{2+} mole ratios in Cu^{2+} solution and the fitted linear equation (a) 1:1, (b) 2:1, (c) 3:1, (d) 4:1, and (e) 5:1.

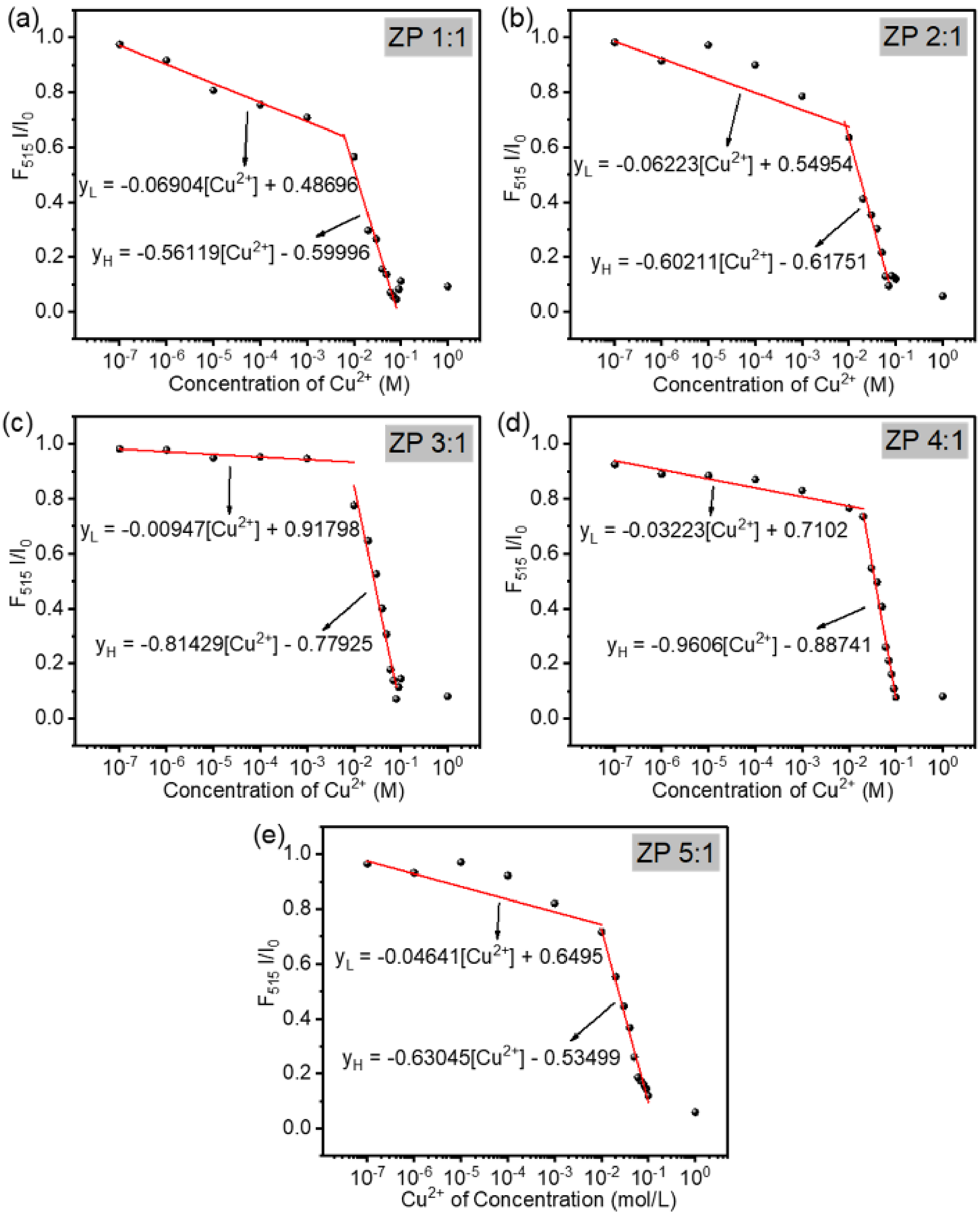

Figure S40. Relative fluorescence intensity of ZP composites prepared using different ligand-to-Zn^{2+} mole ratios in Cu^{2+} solution and the fitted linear equation (a) 1:1, (b) 2:1, (c) 3:1, (d) 4:1, and (e) 5:1.

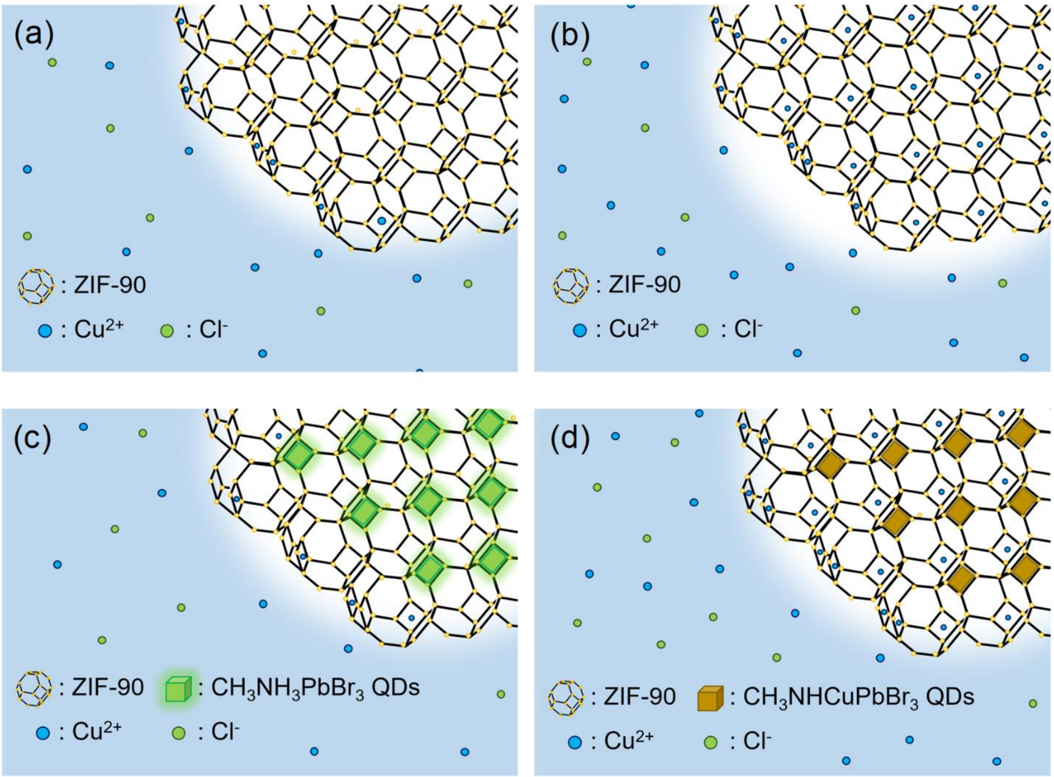

Figure S41. Diffusion kinetics of $CuCl_2$ into ZIF-90 crystals at different $CuCl_2$ concentrations, (a) low and (b) high concentrations. The diffusion kinetics of $CuCl_2$ into ZP composites at different $CuCl_2$ concentrations, (c) low and (d) high concentrations.

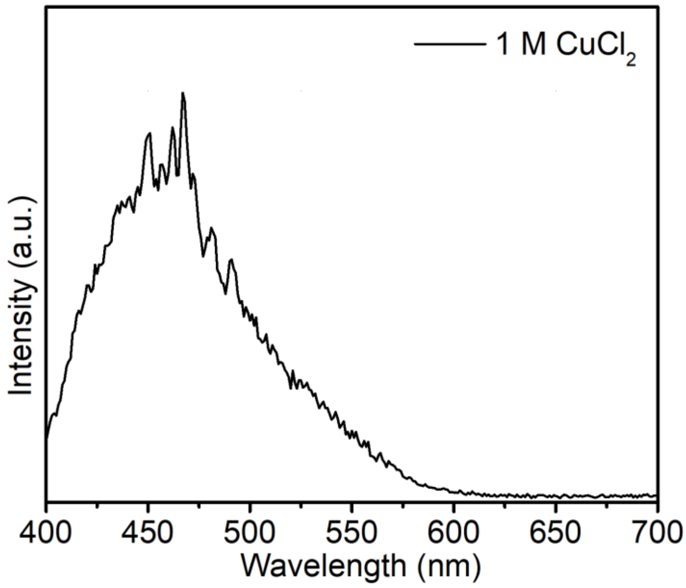

Figure S42. Fluorescence emission spectrum of 1 M $CuCl_2$ aqueous solution.

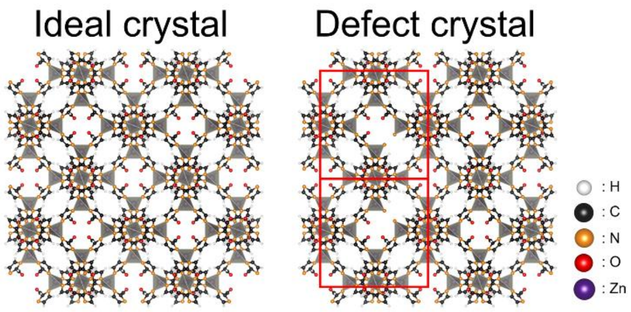

Figure S43. ZIF-90 structure ideal crystal and defect crystal.

References:

(1) Zhang, D.; Xu, Y.; Liu, Q.; Xia, Z. Encapsulation of $CH_3NH_3PbBr_3$ Perovskite Quantum Dots in MOF-5 Microcrystals as a Stable Platform for Temperature and Aqueous Heavy Metal Ion Detection. *Inorg. Chem.* **2018**, *57*, 4613–4619.

(2) Chen, R.; Zhan, K.; Xing, Y.; Wang, Z.; Zhu, Y.; Yan, J.; Liu, B.; Chen, Y. Ultrastable $MAPbBr_3$@ZIF-8$Co_{5\%}$ as a Novel Fluorescence Probe for Highly Selectively Sensing Fe^{3+} in Water. *J. Lumin.* **2021**, *235*, 118056.

(3) Chen, R.; Zhan, K.; Wu, Y.; Zhu, Y.; Yan, J.; Liu, B.; Chen, Y. A Novel Fluorescence Probe Based $MAPbBr_3$@ZIF-8 for Detecting Hypochlorite in Water Samples. *Microchem. J.* **2022**, *172*, 106924.

(4) Jiang, X.; Zhang, J.; Fan, R.; Zhou, X.; Zhu, K.; Yang, Y. Multiple Interpenetrating Metal–Organic Frameworks with Channel-Size-Dependent Behavior for Selective Gossypol Detection and Perovskite Quantum Dot Encapsulation, *ACS Appl. Mater. Interfaces* **2022**, *14*, 49945–49956.

(5) Xiang, X.; Li, J.; Xue, J.; Fu, Z. Preparation of $CH_3NH_3PbBr_3$ Perovskites Encapsulated in ZIF-8 with Improved Stability and Their Application in Fluorimetry and Information Encryption. *Langmuir* **2023**, *39*, 5315–5322.

(6) George, J. K.; Gayathri, K.; Pasha, A.; Mohan, S.; Balakrishna, R. G. Binding of $CsPbBr_3$ Nanocrystals to MOF-5 for the Detection of Cadmium Ions in Aqueous Media. *ACS Appl. Nano Mater.* **2023**, *6*, 9464–9474.

(7) Liu, C.; Yan, B. Luminescent Zinc Metal–Organic Framework (ZIF-90) for Sensing Metal Ions, Anions and Small Molecules, *Photochem. Photobiol. Sci.* **2015**, *14*, 1644–1650.
